# A Genome-Wide Association Study of Psoriasis and Psoriatic Arthritis Identifies New Disease Loci

**DOI:** 10.1371/journal.pgen.1000041

**Published:** 2008-04-04

**Authors:** Ying Liu, Cynthia Helms, Wilson Liao, Lisa C. Zaba, Shenghui Duan, Jennifer Gardner, Carol Wise, Andrew Miner, M. J. Malloy, Clive R. Pullinger, John P. Kane, Scott Saccone, Jane Worthington, Ian Bruce, Pui–Yan Kwok, Alan Menter, James Krueger, Anne Barton, Nancy L. Saccone, Anne M. Bowcock

**Affiliations:** 1 Division of Human Genetics, Department of Genetics, Washington University School of Medicine, St. Louis, Missouri, United States of America; 2 Cardiovascular Research Institute and Center for Human Genetics, University of California San Francisco, San Francisco, California, United States of America; 3 Laboratory for Investigative Dermatology, The Rockefeller University, New York, New York, United States of America; 4 Seay Center for Musculoskeletal Research, Texas Scottish Rite Hospital for Children, Dallas, Texas, United States of America; 5 Department of Psychiatry, Washington University School of Medicine, St. Louis, Missouri, United States of America; 6 Arc Epidemiology Research Unit, University of Manchester, Manchester, United Kingdom; 7 Department of Internal Medicine, Division of Dermatology, Baylor University Medical Center, Dallas, Texas, United States of America; Baylor College of Medicine, United States of America

## Abstract

A genome-wide association study was performed to identify genetic factors involved in susceptibility to psoriasis (PS) and psoriatic arthritis (PSA), inflammatory diseases of the skin and joints in humans. 223 PS cases (including 91 with PSA) were genotyped with 311,398 single nucleotide polymorphisms (SNPs), and results were compared with those from 519 Northern European controls. Replications were performed with an independent cohort of 577 PS cases and 737 controls from the U.S., and 576 PSA patients and 480 controls from the U.K.. Strongest associations were with the class I region of the major histocompatibility complex (MHC). The most highly associated SNP was rs10484554, which lies 34.7 kb upstream from HLA-C (P = 7.8×10^−11^, GWA scan; P = 1.8×10^−30^, replication; P = 1.8×10^−39^, combined; U.K. PSA: P = 6.9×10^−11^). However, rs2395029 encoding the G2V polymorphism within the class I gene HCP5 (combined P = 2.13×10^−26^ in U.S. cases) yielded the highest ORs with both PS and PSA (4.1 and 3.2 respectively). This variant is associated with low viral set point following HIV infection and its effect is independent of rs10484554. We replicated the previously reported association with interleukin 23 receptor and interleukin 12B (IL12B) polymorphisms in PS and PSA cohorts (IL23R: rs11209026, U.S. PS, P = 1.4×10^−4^; U.K. PSA: P = 8.0×10^−4^; IL12B:rs6887695, U.S. PS, P = 5×10^−5^ and U.K. PSA, P = 1.3×10^−3^) and detected an independent association in the IL23R region with a SNP 4 kb upstream from IL12RB2 (P = 0.001). Novel associations replicated in the U.S. PS cohort included the region harboring lipoma HMGIC fusion partner (LHFP) and conserved oligomeric golgi complex component 6 (COG6) genes on chromosome 13q13 (combined P = 2×10^−6^ for rs7993214; OR = 0.71), the late cornified envelope gene cluster (LCE) from the Epidermal Differentiation Complex (PSORS4) (combined P = 6.2×10^−5^ for rs6701216; OR 1.45) and a region of LD at 15q21 (combined P = 2.9×10^−5^ for rs3803369; OR = 1.43). This region is of interest because it harbors ubiquitin-specific protease-8 whose processed pseudogene lies upstream from HLA-C. This region of 15q21 also harbors the gene for SPPL2A (signal peptide peptidase like 2a) which activates tumor necrosis factor alpha by cleavage, triggering the expression of IL12 in human dendritic cells. We also identified a novel PSA (and potentially PS) locus on chromosome 4q27. This region harbors the interleukin 2 (IL2) and interleukin 21 (IL21) genes and was recently shown to be associated with four autoimmune diseases (Celiac disease, Type 1 diabetes, Grave's disease and Rheumatoid Arthritis).

## Author Summary

Psoriasis (PS) and psoriatic arthritis (PSA) are common inflammatory diseases of humans affecting the skin and joints. Approximately 2% of Europeans are affected with PS, and ∼10–30% of patients develop PSA. Genetic variation in the MHC (multiple histocompatibility locus antigen cluster) increases risk of developing PS. However, only ∼10% of individuals with this risk factor develop PS, indicating that other genetic effects and environmental triggers are important. Recent approaches using a case/control approach and genome wide association studies with DNA markers known as SNPs (single nucleotide polymorphisms) have been fruitful in identifying genetic factors for common diseases. This study describes the first large scale genome wide scan for additional PS and PSA susceptibility genes using 233 cases and 519 controls. It revealed that the MHC is truly the most important risk factor for PS and that it plays a very major role in PSA, confirmed recently identified associations with interleukin 23 receptor and interleukin 12B in both PS and PSA, and identified new associations. These include a region on chromosome 4q27 that contains genes for interleukin 2 and interleukin 21 that has been recently implicated in other autoimmune diseases, and seven additional regions that include chromosome 13q13 and 15q21.

## Introduction

Psoriasis (PS) is a chronic inflammatory disease of the skin affecting 2–3% of the population [1]. Approximately 25% of patients also develop psoriatic arthritis (PSA), a common, debilitating auto-immune disease belonging to the family of spondyloarthritides [2],[3]. The recurrence risk (λ_S_) of PSA is high, and estimates of 27–47 have been proposed [4],[5]. This is much higher than the estimated λ_S_ of PS which is estimated to be between 4 and 11[6].

PS and PSA are interrelated disorders, and the prevalence of PS is 19 times higher among first degree relatives of probands with PSA compared with the general population [7]. The pathogenesis of PS and PSA is complex, involving both genetic and environmental risk factors. Strong association of PS with the MHC class I region (PSORS1 or psoriasis susceptibility locus 1) was demonstrated in the 1970s [8] and has been confirmed in numerous subsequent studies [9]–[11]. However, the genetics of PSA is not as clear-cut and association with alleles of the HLA class I region is not reported to be as strong with PSA as with PS [7]. Hence, it has not been clear if PSA is a clinical phenotype that is distinct from PS without psoriatic arthritis and if is due to different predisposing genetic factors.

A number of regions in the genome have been reported to be associated with PS [1], [12]–[14], and some have been convincingly replicated. This includes the 3'UTR of interleukin 12B (IL12B) [15],[16] and two non-synonymous SNPs of interleukin 23 receptor (IL23R) [16]. One of these (R381Q) was also shown to be associated with Crohn's disease [17]. However, together with the PSORS1 locus, the combined effect of these loci is unable to account entirely for genetic susceptibility to PS.

In order to systematically search for other susceptibility loci, we undertook a genome wide association (GWA) scan to identify genetic factors predisposing to PS and PSA. Besides detecting strong association with the HLA class I region in the combined and PSA cohort, and replicating the recently reported associations with IL23R and IL12B, we identified a number of novel associations. These include a region on chromosome 13q13 harboring LHFP and COG6, a region on chromosome 15q21 harboring USP8-SPPL2A-TNFAIP8L3, association with the LCE cluster of genes on chromosome 1q21 from the PSORS4 locus, and a region of chromosome 4q27 recently reported to be associated with several other autoimmune diseases and associated with PSA and potentially PS.

## Results/Discussion

For our “discovery” phase, 223 PS cases (132 cases with PS without arthritis and 91 PS cases with arthritis (PSA) were typed on the Illumina HumanHap300 arrays. We compared case data to publicly available genotype data of 519 European controls from the New York Cancer Project[18] collected with the same platform. The number of cases used for this scan is smaller than that used in many recently described genome wide association scans. However, the 91 cases of PSA had at least one first degree relative with PS and were expected to be enriched for genetic factors. Power calculations based on 223 controls and 519 controls indicated that using a threshold of P<5×10^−5^, we had 70% power of detecting a locus with a genotype relative risk (GRR) of 2.0, and over 99% power to detect a locus with a GRR of 3.0 such as the MHC (see below). However, many replicated associations have small GRRs [19] and we had only 10% power of detecting a locus with a GRR of 1.5.

Following the genotyping of samples, stringent quality control measures were implemented. We required that all samples used for the discovery phase passed a 93% genotyping call rate threshold, and that all SNPs passed a 95% call rate threshold. Justification for this threshold is based on the evaluation of empirical distributions (Figures S1 and S2). With sample call rates ≤93%, there was an elevation in observed sample heterozygosity, i.e. deviation from Hardy-Weinberg equilibrium, suggesting possible genotyping errors (e.g., sample contamination or allele drop-out). Likewise, there was a significant discrepancy of missingness between case and control groups when the SNP success rate was <95%.

For the discovery phase, a total of 311,398 SNPs were pruned to 305,983 SNPs after filtering for low call rate, minor allele frequency <0.01 and deviation from Hardy-Weinberg equilibrium (P<0.001). Quality control also led to the removal of 29 samples, leaving 218 cases for further analysis (Table S1). The average genotyping rate in the remaining individuals was 0.995.

To investigate other biases[20] that could be introduced with shared controls, we assessed the median distribution of test statistics using the genomic-control factor λ_GC_ [21]. With a set of 463 ancestry informative SNPs (AIMs), λ_GC_ = 1 indicating no inflation). We also performed analysis on the same set of AIM SNPs with STRUCTURE software[22]. Under the assumption of two population clusters, there was no association between the most likely inferred cluster and case/control status and the average allele frequency difference between clusters was less than 2.5%. These results showed that population substructure is unlikely to be confounding our results. However, analysis of all markers used in the discovery study yielded λ_GC_ = 1.101 before correction (Q-Q plot shown in Figure S3). A similar value was obtained with EIGENSTRAT [23] where λ = 1.107. Examining stratified subsets of cases (PS without arthritis or PSA) with all markers also yielded similar λ values (PS without arthritis: λ_GC_ = 1.07; PSA: λ_GC_ = 1.05). Following adjustment of P values with the genomic control method, λ = 1˜ The discovery P values adjusted by the genomic control method as implemented in PLINK[24] are presented in the tables and figures.

**Figure 1:**
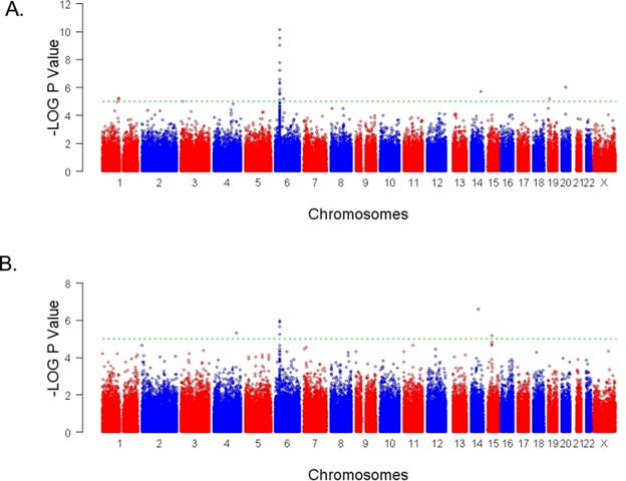
Summary of genome-wide association scan results for all cases and the PSA subgroup. Negative LOG_10_ P values for the Cochran-Armitage test of trend for genome-wide association across the genome and by chromosome are shown. Trend P values were adjusted with the genomic control (GC) method. The spacing between SNPs on the plot is uniform and does not reflect actual physical distances. Adjacent chromosomes are shown in red and then in blue. The horizontal dashed lines display a cutoff of P = 5×10^−5^. A: Results obtained with all cases. B: Results obtained with the subgroup of 91 psoriatic arthritis cases (PSA).

To detect associations, we first performed a preliminary analysis with a Cochran-Armitage trend test [25]. Figure 1 illustrates negative logarithm of the P values obtained across the genome, considering all cases and all controls (Figure 1A) and considering only the 91 PSA cases and all controls (Figure 1B). Results were then rank ordered on the basis of P values. 84 SNPs in 35 genomic regions were associated with P<5×10^−5^; a level that we would informally expect to observe by chance roughly 15 times in this scan given the number of tests performed if all SNPs were independent. A subset of SNPs from 120 regions were investigated further. Criteria for selection included the strength of the discovery P value, particularly when several SNPs from a single region showed evidence for association, a possible biological role of a gene harboring a SNP with some evidence for association, or localization of SNPs with moderate evidence for association to a known psoriasis susceptibility locus (e.g. PSORS4). We also included the previously reported associated SNPs in IL23R and IL12B[16].

An independent cohort of 577 PS cases from the U.S. and 737 U.S. controls were used for the replication stage; 94 of these cases had also been diagnosed with PSA. To examine the potential role of variants upon PSA susceptibility specifically, 576 PSA cases from the UK and 480 controls from the UK were also employed. An alternative genotyping technology (iPlex; Sequenom) was used for the replication phase. The platforms used for the discovery and replication phases gave very similar results: Concordance rates on the basis of 116 samples and 301 SNPs typed with both platforms was 98.74%.

Our 100 top ranked SNPs with any cohort (PS, PS without arthritis, or PSA) are listed in Table S2, to facilitate future attempts to replicate our findings. A total of 289 SNPs, including SNPs from the MHC, and two previously reported associated SNPs within IL12B and IL23R[16] were genotyped in the replication analysis.

### MHC

**Figure 2:**
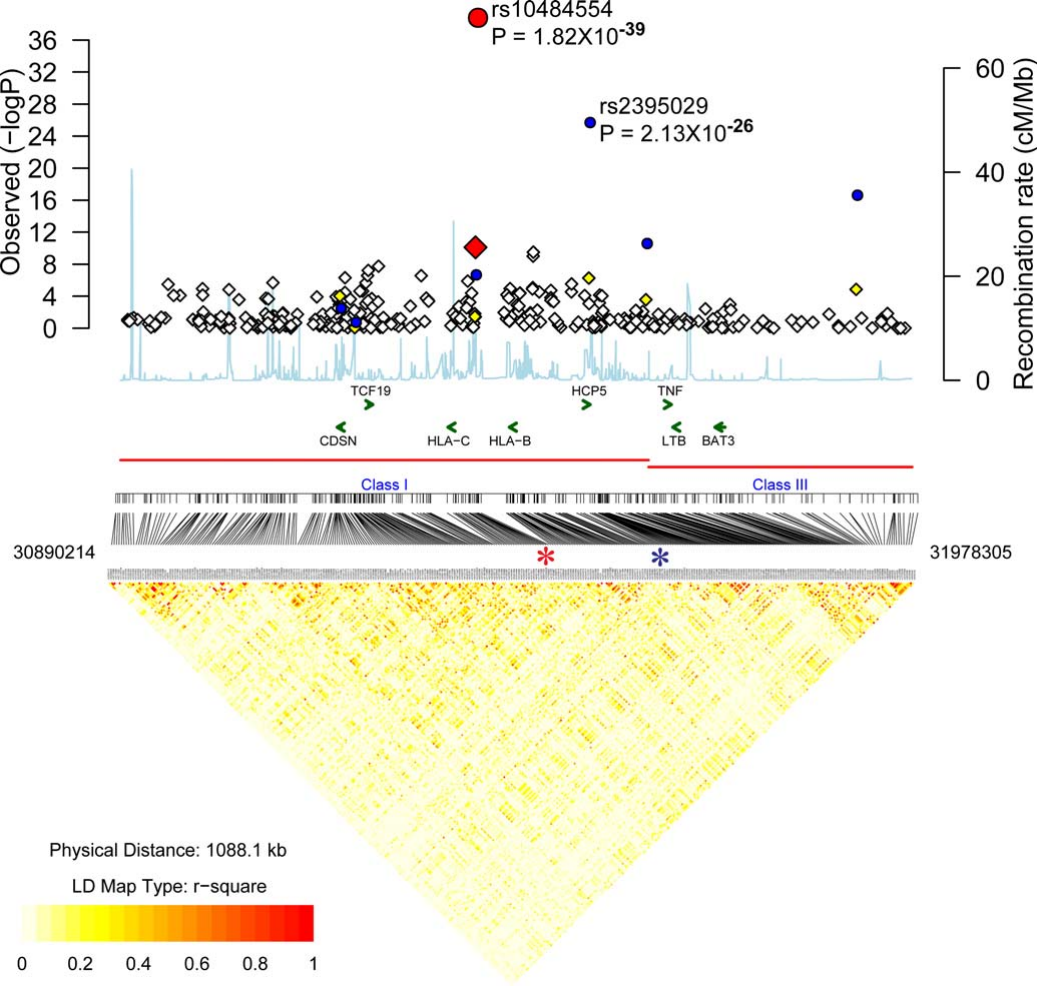
Association localization plots for the MHC following discovery and replication phases. Results for SNPs used in the discovery phase (adjusted for GC) are presented as diamonds. Negative LOG P values are provided on the Y axis. The X axis corresponds to the locations of SNPs. The P value for all samples (original GWA scan plus replication samples) are shown as circles. The P value obtained with the most highly associated SNP (from the original GWA scan plus the replication samples) is shown as a red circle. The SNPs shown as orange diamonds are in r^2^>0.8 (European HapMap CEPH (CEU) samples) with the most significant SNP identified in our study. The recombination rate based on the CEU HapMap is shown in light blue along the x axis (scale on the right). The LD relationship of Illumina discovery SNPs derived from CEU HapMap genotypes are shown below the graph. The most highly associated SNPs are indicated with an asterisk. The green arrows indicate the locations of select genes.

The MHC, and in particular, the HLA class I region, is the only region that has been shown to be consistently associated with PS. The first nine top-ranking SNPs were from the MHC and seven were significant, even when adjusted with the Bonferroni correction for multiple tests (Table S2). Overall, 32 SNPs from the MHC had adjusted P values <5×10^−5^ (Figure 2, Table S2). The most significant association was with rs10484554 (adjusted P = 7.8×10^−11^, GWA scan; P = 5.61×10^−28^, replication; P = 9.772×10^−38^, combined) (Figure 2, Table 1). This SNP lies 34.7 kb upstream from the transcriptional start site of HLA-C. Strongest association with this region is consistent with previous results from our group and others [9]–[11]. The rs10484554*T allele had frequencies of 0.325 in U.S. cases and 0.15 in U.S. controls (OR: 2.8 (95% CI: 2.4–3.3). To determine the relationship of this allele with the classical HLA-C allele strongly associated with psoriasis (HLA-Cw*0602), we investigated the transmission of this allele with classical HLA-C alleles in ∼250 nuclear families with psoriasis that we have reported elsewhere [26]. The rs10484554*T allele was detected on nearly all haplotypes with HLA-Cw*0602 or HLA-Cw*1203 alleles (results not shown), and was also strongly correlated with the previously described highly associated PSORS1 SNP n9*G[26],[27] (rs10456057*G) allele. We have previously shown that SNPs upstream from HLA-C are more strongly correlated with PS than HLA-Cw*0602 is, and that these risk alleles are also correlated with HLA-Cw*1203 [26]. Hence, rs10484554*T may be a good proxy for the PSORS1 variant.

**Table 1:** Summary of association with previously reported PS susceptibility loci (MHC, IL23R and IL12B) in U.S. PS cohort (810 cases, 1256 controls).

Cyto. locn	SNP	Location (hg18)	Gene/region	Disc. P	Disc. P (adj.)	Rep. P	Combined P	Minor allele	Freq. U.S. PS cases	Freq. U.S. Controls	OR US PS combined (95% C.I.)
6p21	rs10484554	31382534	MHC	8.7×10^−12^	7.8×10^−11^	1.82×10−^30^	1.81×10^−39^	T	0.32	0.151	2.8 (2.4, 3.3)
6p21	rs2395029	31539759	MHC	1.4×10^−7^	5.3×10^−7^	2.51×10^−19^	2.13×10^−26^	C	0.12	0.033	4.1 (3.1, 5.3)
1p31	rs11465804	67475114	IL23R	0.4	0.42	ND	0.0072	G	0.044	0.065	0.67 (0.50,0.9)
1p31	rs11209026	67478546	IL23R	0.067	0.081	0.0039	0.00014	T	0.039	0.066	0.56 (0.41,0.76)
1p31	rs12131065	67541594	IL23R	0.0025	0.0039	0.074	0.001	A	0.197	0.243	0.78 (0.66,0.91)
5q33	rs3212217	158687708	IL12B	N.D.	N.D.	0.012	N.D.	G	0.158	0.199	N.D.
5q33	rs6887695	158755223	IL12B	N.D.	N.D.	0.00005	N.D.	C	0.221	0.294	N.D.

Cyto. Locn: Cytogenetic location of SNP; Disc P: Trend P values for the GWA scan; Disc. P (adj.): Genomic control adjusted trend P values for the GWA scan; Rep. P: Trend P values obtained with the U.S. PS replication cohort; Combined P: Trend P values for U.S. discovery and replication cohorts combined ; Freq. U.S. PS cases: Frequency of the minor allele in U.S. psoriasis cases; Freq. U.S. Controls: Frequency of minor allele in the control population. OR: Odds Ratio; C.I.: confidence interval; N.D.: Not Done.

In the case of the U.K. PSA replication samples, rs10484554 was again highly significant (P = 6.86×10^−11^) (Table 2), although the frequency of the rs10484554*T allele exhibited population differences when frequencies in the U.K. and U.S. were compared. In the U.K. the rs10484554*T allele was found at a lower frequency in cases and controls (0.19 and 0.07 respectively; OR: 2.4 (95% CI: 1.8–3.1)).

**Table 2:** Summary of association with previously reported PS susceptibility loci in U.K. PSA cohort (576 cases, 480 controls).

SNP	Gene/Region	Trend P value	Minor allele	Freq. UK PSA cases	Freq. UK PSA controls	OR UK PSA (95% C.I.)
rs10484554	MHC	6.86×10^−11^	T	0.19	0.07	2.4 (1.8, 3.1)
rs2395029	MHC	1.86×10^−10^	C	0.12	0.04	3.2 (2.2, 4.6)
rs11209026	IL23R	0.00083	T	0.043	0.079	0.52 (0.35, 0.77)
rs12131065	IL23R	0.31	A	0.21	0.23	0.89 (0.72, 1.11)
rs3212217	IL12B	N.D.	G	N.D.	N.D.	N.D.
rs6887695	IL12B	0.0013	C	0.213	0.28	0.69 (0.56, 0.85)

UK PSA: U.K. Psoriasis cases with arthritis; OR: Odds Ratio; C.I.: confidence interval; N.D.: Not Done.

A second SNP from the HLA class I region lying between MICA and MICB (rs2395029) was highly associated with PS and PSA. This SNP results in the G2V polymorphism of the class I gene HCP5 (HLA complex P5) which encodes an endogenous retroviral element. For this SNP, PS was associated with a combined P = 2.13×10^−26^ in the U.S. cohort and 1.86×10^−10^ in the U.K. PSA cohort (Table 1, Table 2). The OR of the rs2395029*C allele with both PS and PSA was higher than with any other SNP tested (4.1 and 3.2 with PS and PSA respectively). This allele was found at a frequency of ∼0.12 in cases and 0.04 in controls and did not exhibit the population frequency differences of rs10484554. The LD relationship between rs2395029 and rs10484554 is not strong (r^2^ = 0.33 in European CEPH HapMap samples and r^2^ = 0.23 in our U.S. case/control cohort). Conditioning upon rs10484554, the P value for rs2395029 was still significant (P = 7×10^−10^), hence effects from this SNP are likely to be independent.

HCP5 is expressed primarily in cells of the immune system such as spleen, blood and thymus (http://smd-www.stanford.edu/), consistent with a potential role in autoimmunity. This allele was recently shown to explain 9.6% of the total variation in viral set point following HIV-1 infection[28]. This is of interest, since psoriasis can be triggered by infection with HIV and other viruses. Hence, it is possible that HCP5-C carriers mount a strong immune reaction to viral infection, but that in genetically susceptible individuals, this reaction leads to excessive inflammation in skin and joints. Overall, our observations indicate that MHC class I region SNPs are more highly associated with both PS and PSA than any other SNPs.

### IL23R associations

A recent global association scan using a set of pooled PS samples and controls against a set of 25,215 genecentric SNPs confirmed a previously reported association with IL12B (rs3212227 in its 3′ UTR) [15] and identified a second region of association 60 kb upstream from its mRNA start site (rs6887695) [16]. An analysis of additional genes encoding components of the IL12B pathway lead to the identification of associations with Il23R (R381Q: rs11209026 and L310P: rs7530511) [16]. These SNPs were proposed to mark a common psoriasis-associated haplotype. Rs11209026 is also the SNP within IL23R reported to be associated with Crohn's disease[17].

In our discovery cohort, the most significant association in the IL23R interval was obtained with a different SNP from that described previously as being associated with PS (rs11209026). This SNP, where P = 0.0039 in the discovery cohort (Table 1), has not previously been reported to be associated with PS. The LD relationship between rs12131065 and the previously associated rs11209026 SNP is low (r^2 = ^0.031 in HapMap CEPH European samples; 0.009 in cases; 0.026 in controls). Conditioning upon rs11209026, the P value for rs11209026 was 0.013. Hence, effects from this SNP may be independent of rs11209026 and its association with PS should be investigated in other cohorts.

**Figure 3 pgen-1000041-g003:**
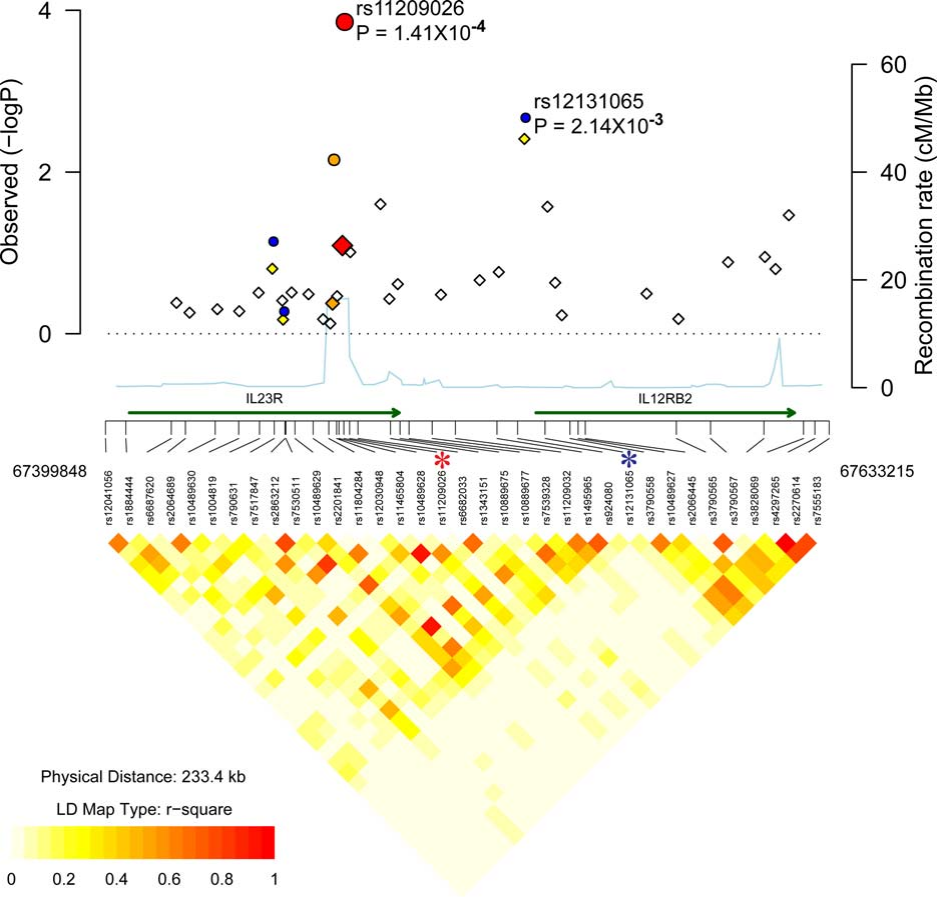
Association localization plots for the ILI23R region on chromosome 1. Symbols are the same as those used in Figure 2. SNPs indicated with an asterisk are rs11465804, rs11209026 (R381Q) and rs12131065.

SNP rs12131065 lies downstream from IL23R (63 kb from rs11209026) and 4.041 kb upstream from the gene for interleukin 12 receptor B2 (IL12RB2) (Figure 3). IL12RB2 is involved in IL12 dependent signaling, is upregulated by gamma interferon in T_h_1 cells and plays a role in T_h_1 differentiation[29]. Association with a SNP closer to IL12RB2 than IL23R is of interest since animals where IL12RB2 is inactivated develop autoimmune disease[30].

Association with the previously reported IL23R associated SNP rs11209026 in the discovery cohort was not significant (adjusted P = 0.081). Genotyping of rs11209026 and rs12131065 in the U.S. replication cohort yielded combined P values of 1.4×10^−4^ and 0.001 respectively (Table 1) consistent with replication of this locus with respect to previous studies. In the case of these two SNPs, the protective T and A alleles were found at frequencies of ∼0.04 and 0.2 in cases versus ∼0.07 and ∼0.24 of controls respectively. In the U.K. replication PSA cohort, association with rs11209026 was consistent with replication (P = 8.3×10^−4^), with the rs11209026*T allele being found at frequencies of ∼0.04 in cases and ∼0.08 in controls (Table 2).

### IL12B Associations

Although the associated IL12B SNPs rs3212227 and rs6887695 were not interrogated by the Illumina screening panel of SNPs used here, typing of these SNPs in our replication U.S. case/control cohorts yielded P values of 0.021 and 5×10^−5^ and replicated previously reported associations (Table 1). In the U.K. PSA cohort, association with rs6887695 was also consistent with replication (P = 0.0013) (OR: 0.69; 95% CI: 0.56–0.85)) (Table 2).

### Novel Psoriasis Loci

**Figure 4:**
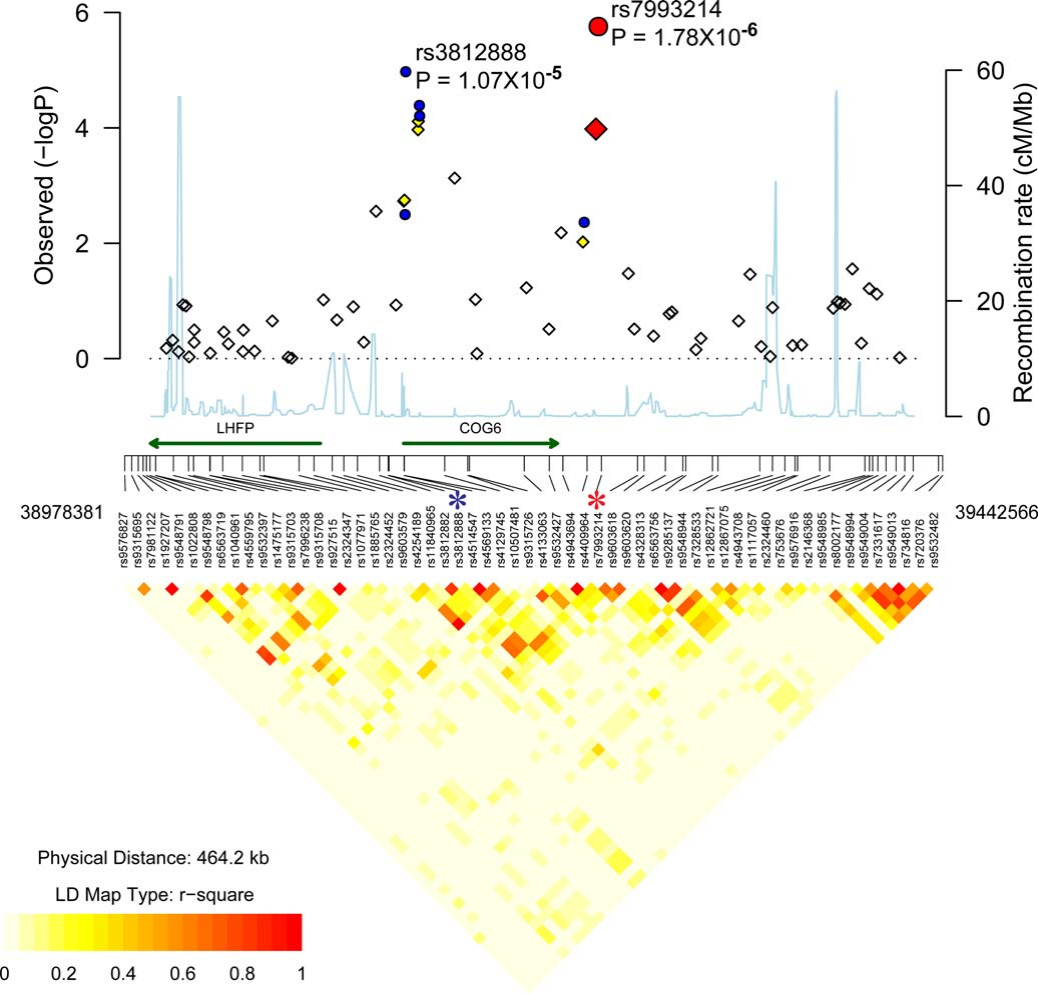
Association localization plots for novel replicated region on chromosome 13. Symbols are the same as those used in Figure 2. SNPs indicated with an asterisk are rs3812888 and rs7993214.

In the discovery cohort, there were four SNPs from 13q13 where P<5×10^−5^ (adjusted P<2×10^−4^). These were: rs1186468, rs4514547, rs4569133 and rs7993214 (Table S2). These SNPs lie within a region on chromosome13q13 that encodes the conserved oligomeric golgi complex component 6 (COG6) gene and a lipoma HMGIC fusion partner (LHFP)[31]. Three of the top associated SNPs were tested in the U.S. replication cohort, and all showed evidence of replication at P<0.05 (Table 3, Figure 4). Results were most significant with rs7993214 (adjusted P  = 10^−4^, GWA scan; P = 0.0033, replication; P = 2×10^−6^, combined). Rs3812888, (adjusted P = 0.0017, GWA scan; P = 4×10^−4^, replication; P = 10^−5^, combined) was the only SNP where replication results would remain significant following the stringent Bonferroni correction for multiple tests (P = 0.048). The OR of the rs3812888*C allele was 1.38 (95% CI: 1.15–1.66). The rs3812888*C allele was found at frequencies of 0.43 in cases and 0.35 in controls.

**Figure 5:**
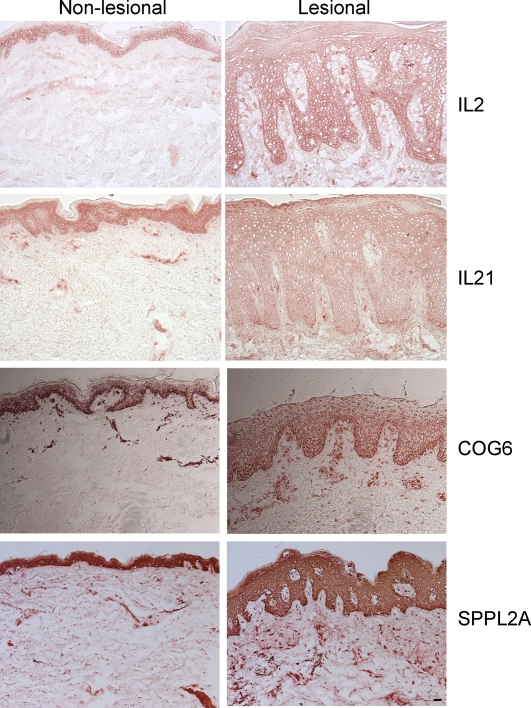
Immunostaining of normal, non-lesional and lesional skin for IL2, IL21, COG6 and SPPL2A proteins. The size bar is 100 micrometers.

COG6 is a component of the conserved oligomeric golgi (COG) complex and is involved in intracellular transport and glycoprotein modification[32]. The glycosylation pathways in the golgi apparatus must be intact for protein secretion to continue unabated. In *C. elegans*, a COG complex is required to glycosylate an ADAM protease (a disintegrin and metalloprotease)[33]. In humans, variants within some ADAM genes lead to inflammatory diseases. For example, ADAM33 is an asthma susceptibility gene whose catalytic domain undergoes glycosylation[34]. Recent genetic studies suggest that ADAM33 is a psoriasis susceptibility gene as well [35]. Hence, COG6 could be involved in glycosylation of ADAM33 or other ADAM proteases. Staining of skin sections with a COG6 antibody revealed cytoplasmic staining in the epidermis as well as strong T-cell staining (Figure 5). There was variable expression of the protein in non-lesional skin samples, but there was uniformly strong expression in all lesional sections. Very little is known about LHFP. It is a subset of the superfamily of tetraspan transmembrane protein encoding gene. Expression analysis from SOURCE (http://smd-www.stanford.edu/) indicates that highest levels are found in the ear and spinal cord.

**Table 3:** Potentially novel loci from GWA scan.

Cytogenet. location	SNP	Location (hg18)	Gene/region	Disc. P	Disc. P (adj.)	Rep. P	Combined P	Minor allele	Freq cases	Freq. Controls	OR (95% C.I.)
1q21	rs6701216	151045150	EDC	0.0034	0.0053	0.0069	0.00005	T	0.174	0.127	1.45 (1.21,1.75)
2p11	rs2164807	85816062	GNLY-ATOH8	0.00084	0.0015	0.0039	0.000016	G	0.467	0.394	1.35 (1.18,1.54)
2p12	rs11126740	79754603	CTNNA2	0.0018	0.0018	0.019	0.00014	T	0.311	0.37	0.77 (0.67,0.88)
3q13	rs6804331	105237077	Gene desert	0.00073	0.0013	0.0455	0.0003	C	0.445	0.381	1.30 (1.13,1.50)
13q13	rs3812888	39128294	COG6	0.001	0.0017	4×10^−4^	0.00001	C	0.43	0.35	1.38 (1.15,1.66)
13q13	rs7993214	39248912	COG6	4.7×10^−5^	0.0001	0.0033	2×10^−6^	T	0.279	0.351	0.71 (0.62,0.82)
14q32	rs2282276	94730882	CLMN	0.0096	0.014	0.047	0.0031	G	0.099	0.073	1.40 (1.12,1.76)
15q21	rs4775912	49068271	USP8-TNFAIP8L3	0.00034	0.00065	0.0136	5.6×10^−5^	G	0.194	0.146	1.41 (1.19, 1.67)
15q21	rs3803369	49163121	USP8-TNFAIP8L3	0.00012	0.00025	0.0138	2.9×10^−5^	A	0.195	0.145	1.43 (1.21,1.69)

Trend P values from discovery, replication and combined analyses for U.S. PS samples (810 cases, 1256 controls) are shown. The minor allele, its frequency in cases and controls and its odds ratios and 95% C.I.s from the combined data-set are also shown. Abbreviations are described in footnotes to Table 1.

**Figure 6:**
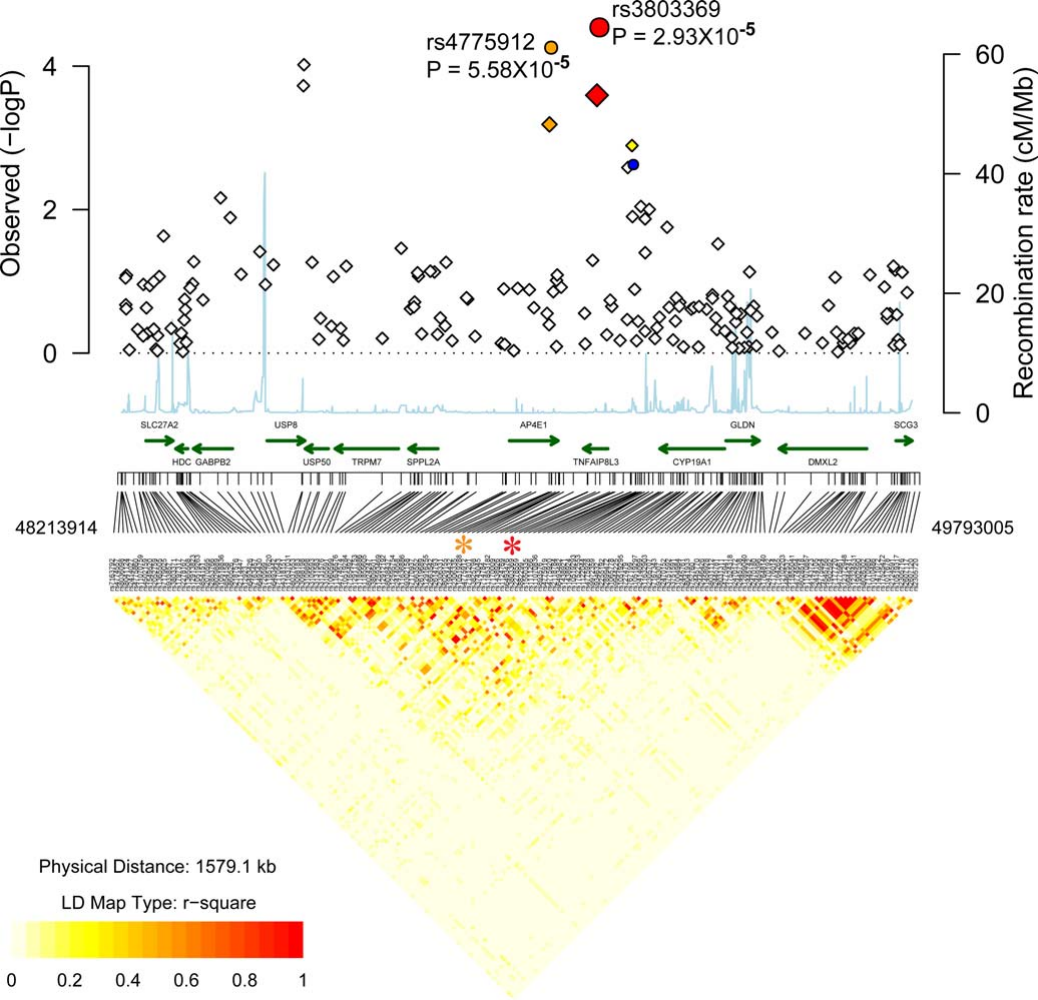
Association localization plots for novel replicated region on chromosome 15. Symbols are the same as those used in Figure 2. SNPs indicated with an asterisk are rs4775912 and rs3803369.

When the PSA discovery cohort (n = 91) was analyzed separately, four SNPs from a region on chromosome 15q21 between ubiquitin specific protease 8 (USP8) and tumor necrosis factor, alpha-induced protein 8-like 3 (TNFAIP8L3) were associated with P<5×10^−5^ (Table S2). In the case of the most highly associated SNP (rs4775919), adjusted P = 6.7×10^−6^. Following replication genotyping, this and rs3803369 were associated with PS with P values consistent with replication (for rs3803369, adjusted P = 2.5×10^−4^, GWA scan; P = 0.013, replication; P = 2.9×10^−5^ combined; Table S2, Table 3, Figure 6). The rs3803369*A allele was found at a frequencies of ∼0.2 in cases and 0.15 in controls (OR 1.43, 95% CI: 1.21–1.69). Other genes in this region include the transient receptor potential melastatin 7 (TRPM7)[36], signal peptide peptidase like 2a (SPPL2A)[37] and AP4E1, a member of the heterotetrameric adaptor protein (AP) complexes (Figure 6). TNFAIP8L3 is a novel protein. It harbors a domain (DUF758) that is found in several proteins induced by tumor necrosis factor alpha (TNFA), but its function is unknown. However, the most plausible candidate is SPPL2A that catalyzes the intramembrane cleavage of TNFA, triggering the expression of IL12 by activated human dendritic cells[38]. Staining of skin sections with an SPPL2A antibody (Figure 5) revealed profound staining of the epidermis, and staining of some dermal cells in both lesional and non-lesional skin.

This region is also of interest however, because a processed pseudogene for one of the genes in this region (USP8) is found upstream from HLA-C[27]. As discussed earlier, this region is one that is most likely to harbor the PSORS1 variant. To ensure that our observations were not due to cross-hybridization of chromosome 15q21 SNPs with PSORS1 SNPs, we investigated alignment of genes from this region of chromosome 15q21 with the remainder of genome. We did not detect any significant identity with any other region, including the MHC. This, and the fact that chromosome 15 associated SNPs are in Hardy-Weinberg equilibrium indicate that our results are unlikely to be artifactual and due to amplification of PSORS1 sequences. The similarity between the PSORS1 and 15q21 variants and their biological consequences need to be investigated further since it may provide important insights into the nature of the PSORS1 variant. However, it is worth noting that our PSA cases which provided strongest evidence for association with 15q21 all had at least one first degree relative with PS, and association with this locus may be harder to detect in “sporadic” cases.

We also observed association of PS with a region of the Epidermal Differentiation Complex (EDC), which harbors a previously established psoriasis locus (PSORS4)[39]. In this instance, rs6701216 yielded a combined P = 6.2×10^−5^ (OR 1.45) (Table 3, Figure S4). This SNP lies within the late cornified envelope 1C gene (LCE1C)[40], and is one of a family of genes that are transcribed very late in epidermal differentiation. Our localization of PSORS4 to the LCE region is of interest, and its refinement will be described in detail elsewhere (Robarge et al., in preparation).

There were two other regions selected for follow-up where P values were <0.05 in the PS replication cohort, and where evidence for association increased in the combined cohort (Table 3). One was an intergenic region located between granulysin (GNLY) and atonal (ATOH) on chromosome 2p11. The most highly associated SNP was rs2164807 (adjusted P = 0.0015, GWA scan; P = 0.0039, replication; P = 1.6×10^−5^ combined). For this SNP, the G allele was found at a frequency of ∼0.47 in cases and ∼0.39 in controls (OR: 1.35, 95% CI: 1.18–1.54). GNLY (Protein NKG5, Lymphokine LAG-2) is of considerable interest with respect to psoriasis. It is present in cytotoxic granules of cytotoxic T lymphocytes and natural killer cells, and is released upon antigen stimulation[41]. It has been shown to have antimicrobial activity against a broad range of microbes including Gram-positive and Gram-negative bacteria, fungi, and parasites including *M. tuberculosis* and other organisms. Priming of granulysin in CD4 is dysregulated in the CD4+ T cells of HIV-infected patients[42].

Other genes that should be evaluated in additional PS cohorts on the basis of replication P values <0.05, and increased significance in combined cohorts (Table 3) are calponin-like transmembrane domain protein (CLMN)[43], the gene for the catenin member, CTNNA2[44] and a gene desert on chromosome 3q13.

### Association with Chromosome 4q27: A Recently Identified Autoimmune Locus

In the discovery cohort there were 3 SNPs from chromosome 4q27 with P<5×10^−5^ (adjusted P<10^−4^). These were rs13151961, rs6822844 and rs6840978 (Table S2, Figure S6). The most significant of these was rs13151961, where adjusted P = 4×10^−5^ (Table 4). Association of this region with PSA was confirmed in an independent cohort of patients from the UK (for rs13151961, P = 0.003, Table 4), where the frequency of the associated T allele was ∼0.25 in cases and ∼0.31 in controls (OR: 0.64; 95% CI: 0.49–0.84). Association with additional SNPs in high LD with rs13151961 (rs6840978 and rs6822844), was also replicated in this cohort. Although association could not be replicated in our U.S. cohort with a case/control approach, the trend in allele frequencies in cases versus controls was similar to that seen in the U.K. cohort (for rs13151961 the frequency of the T allele was ∼0.26 in U.S. cases and ∼0.29 in our U.S. controls, Table 4).

**Table 4:** Association results at chromosome 4q27 in PSA and PS cohorts (U.S.: 810 PS cases, 1256 U.S. controls; U.K.: 576 PSA cases, 480 controls).

SNP	Location (hg18)	Disc. P	Disc. P (adj.)	UK PSA P value	Minor allele	Freq UK PSA cases	Freq UK controls	OR UK (95% CI)	Rep. US PS P value	Freq US PS. cases	Freq US controls	OR US PS cases.(95% CI)
rs13151961	123334952	1.6×10^−5^	3.98×10^−5^	0.0016	G	0.121	0.175	0.64(0.49,0.84)	0.17	0.138	0.158	0.86 (0.71,1.04)
rs7684187	123560609	1.6×10^−4^	3.3×10^−4^	0.001	T	0.247	0.313	0.72 (0.59,0.87)	0.52	0.259	0.298	0.82 (0.71,0.95)
rs6822844	123728871	4.3×10^−5^	9.6×10^−5^	0.008	A	0.155	0.203	0.72 (0.56,0.92)	0.29	0.143	0.164	0.85 (0.71,1.03)
rs6840978	123774157	2.9×10^−5^	6.6×10^−5^	0.013	A	0.192	0.236	0.77 (0.62,0.95)	0.87	0.172	0.204	0.81 (0.68,0.96)

The minor allele, its frequency in cases and controls and its odds ratios and 95% C.I.s from the combined dataset are also shown. Abbreviations are described in footnotes to Table 1.

A recent study from the Wellcome Trust Case Control Consortium [19] identified this 4q27 region in a search for risk factors for type 1 diabetes (T1D). In a follow-up study [45], some support for this association with T1D was provided. In the latter study, association of this region with Grave's disease (GD) was also tested, and some evidence for association with the complementary allele of rs17388568 to that seen in T1D was obtained. The same region was also reported to be associated with Celiac disease (CeD) [46]. Recent evidence is also provided for a role for this region in rheumatoid arthritis and T1D from the Netherlands[47]. In that study the rs6822844*A allele was reported to be a perfect proxy for a haplotype that is highly associated with autoimmune diseases[47] with frequencies of 0.13 in cases versus 0.19 in N. European controls.

The risk alleles of rs13151961, rs13119723, rs6822844 and rs6840978 associated with PS and PSA in the current study are similar to those reported for CeD [46]. Overall, the risk alleles/haplotype of GD, CeD, PS and PSA appear to be the same, and of similar frequency. For example the frequency of the rs6822844*A allele is 0.14 in U.S. PS patients, ∼0.16 in U.K. PSA patients, 0.14 in RA patients [47], ∼0.13 in T1D patients from the Netherlands[47], 0.12 in Celiac Disease patients from the Netherlands[46], and ∼0.14 in Irish CD patients[46]. This contrasts with frequencies of 0.19–0.20 in European control populations[46] [47]. Although the frequency of the rs6822844*A allele was 0.14 in our PS cases and hence similar to frequencies seen in CeD, RA and T1D cases, its frequency in our combined cohort of U.S. controls was 0.16 (Table 4). This is lower than that reported for European controls. However, it has been previously reported that geographic variability exists at this locus across Europe [46]. Our U.S. “European” controls are likely to be more disparate in origin, and are likely to account for our inability to obtain significant evidence for association with PS and 4q27.

To explore association of this region in our cohort of U.S. PS cases, without the possible confounding influence of subtle geographic variability at this locus in Europeans, we performed family based association tests in our 242 psoriasis nuclear families which are described elsewhere[26],[48]. This approach provided evidence for replication of association of PS with rs6822844 and rs6840978 (PDT P = 0.029 and 0.007 respectively). For these SNPs, the over-transmitted rs6822844*G and rs6840978*C alleles were also the risk allele from case/control studies. Haplotype studies in families also revealed association with the rs6822844*G/rs6840978*C haplotype (multipoint TDT P = 0.006). Hence, our findings support chromosome 4q27 as harboring a variant/haplotype for PSA and PS.

As reported elsewhere the 4q27 locus that contains these associated SNPs corresponds to two closely correlated ∼439 kb and ∼40 kb haplotype blocks [46]. This extensive LD makes it very difficult to determine the predisposing variant. Chromosome 4q is also the location of PSORS3, which is generally placed slightly more distally [49]. However, the locus identified here may contribute in part to the previous observations of linkage. The long region of LD at chromosome 4q27 contains several genes [46]: Testis nuclear RNA-binding protein (TENR), a gene encoding a protein of unknown function (KIAA1109), and genes encoding the interleukin-2 (IL2) and interleukin-21 (IL21) cytokines. TENR is expressed primarily in testis and KIAA1109 transcripts are ubiquitous, hence their roles in autoimmunity are not particularly compelling. However, IL2 and IL21 are of particular interest with respect to PS. IL2 is considered to be a pathogenic cytokine for PS[50], and blockade of the IL2 receptor with therapeutic antibodies has induced disease resolution in some cases[51]. IL2 is a survival factor for T cells and promotes the differentiation of cytotoxic T-lymphocytes and NK cells. Both of these cell types are present in psoriasis lesions. Moreover, many IL2 receptor (IL2R) positive T-cells that fit the phenotypic definition of regulatory T cells (T_regs_) are also present in psoriasis lesions. IL-2 may influence how a common precursor T-cell differentiates into either a T_reg_ or a T_h_17 T-cell, since addition of IL-2 has been shown to suppress the differentiation of T_h_17 T-cells in mice[52]. IL-2 antibodies stain normal epidermis and psoriatic epidermis, with generally lower staining in the dermis (Figure 5). The pattern of staining appears to be to dendritic cells (DCs) which are likely to be epidermal Langerhans cells. This pattern of IL-2 staining is probably due to DC activation and upregulation of IL2R. Cells with IL2 receptors include T-cells, B-cells, NK-cells, and dendritic cells.

The epidermal staining for IL-21 is much lower than for IL-2 (Figure 5) and appears to be mainly on dendritic cells in the superficial dermis. IL-21 is a product of activated T cells (under conditions of T_h_17 polarization). It then acts in an autocrine or paracrine fashion on T-cells to up-regulate expression of the IL23 receptor which has already been implicated in psoriasis pathogenesis. IL23R sensitizes cells to IL-23 which stimulates IL17 synthesis and/or prolongs the survival of T_h_17 cells [53]. Blocking IL21 reduces the progression of lupus erythematosus [54] which is one of the autoimmune diseases that is now being considered as a “T_h_17” mediated disease. Therefore IL21 may play a role in T_h_17 formation in this and other autoimmune diseases where these cells are pathogenic.

Extensive resequencing of IL2 coding and flanking regions has already been performed in T1D samples and no coding or obvious regulatory/splice variants were identified [45]. As stated previously, this region needs to be resequenced thoroughly followed by comprehensive genotyping in larger numbers of samples to identify the autoimmune associated variant/s [45].

### Conclusions

The observed associations in the current study are of interest for several reasons. It is noteworthy that the strongest association is with the MHC. Even in PSA, where associations are reportedly less than with PS (without PSA), associations with the class I region appear to be more significant than with any other region. We were also able to replicate previously reported associations with IL12B and IL23R and detected a potentially novel association upstream from IL12RB2. Novel associations within COG6 and the region on chromosome 15q21 harboring USP8 and SPPL2A are of interest. These and other regions reported here are worthy of follow up in other cohorts. Moreover, the association with chromosome 4q27 provides further evidence that this region is a common locus for multiple forms of autoimmune disease.

A recent study reported the IL13/IL4 region from chromosome 5q31 as being associated with PS[55]. Overall, the risk contributed by the MHC, the IL13/IL4 region and the IL23R and IL12B variants was estimated to be 3.83. With the COG6 and chromosome 15q21 loci described here, the risk would be increased. However, PS is a complex disease, and overlapping subsets of risk factors may be sufficient for susceptibility, so that risk effects cannot be computed in an additive manner.

The ability to identify low risk variants for common diseases such as PS and PSA will be limited by the cohort size, and larger numbers of cases and controls will be necessary to identify the majority of genetic factors for these diseases. Moreover, some of the SNPs with borderline discovery P values are also likely to be genetic risk factors for disease. It is worth noting that our discovery P value for the associated IL23R R381Q SNP did not reach significance (P = 0.057, adjusted P = 0.081) although allele frequencies in the discovery dataset revealed a M.A.F. of 0.041 in cases and 0.065 in controls, which is similar to what has been reported in other studies[56]. Additional genome-wide association scans and replication studies are required to identify additional variants and to confirm some of those found in the current study. Such studies include a genome-wide scan for psoriasis variants from the Genome Wide Association Network (GAIN) consortium [57]. Genes such as these are important for determining the pathogenesis of PS and PSA and in identifying novel drug targets for these inflammatory diseases of the skin and joints.

## Materials and Methods

### Subjects

The cohorts for the discovery and replication phases of this study are all of European descent and are described in Table S1. The discovery cohort consisted of 223 Caucasian individuals with PS or PSA from the US. Cases were ascertained through Texas Dermatology (Dallas, TX) and the dermatology clinics at the University of California, San Francisco (UCSF). 89 of the PSA cases had a first degree relative with psoriasis and were members of affected sib pair families, described elsewhere[58]. All except for two of these PSA cases also had PS. These cases were from affected sib pair families with psoriasis and both of these cases had several first degree relatives with PS.

Genotypes of 519 European controls obtained following hybridization to the Illumina HumanHap300 array were from the New York Cancer Project (NYCP) [59] and were downloaded from http://intragen.c2b2.columbia.edu/. These were random controls and there was no specific information about autoimmune/inflammatory disease. Recent large genome-wide association studies using controls of this type have been shown to be successful, leading to only a modest effect on power unless the event of misclassification bias is substantial[19]. Informed consent was obtained from all participants. Protocols were approved by the local institution review boards of all participating institutions. All subjects over 18 years of age gave written informed consent, filled out a clinical questionnaire and received a skin examination by the study dermatologist, who confirmed the diagnosis of plaque PS and graded PS severity. All adults with PSA satisfied the inclusion criteria of having both clinically documented inflammatory synovitis and PS, confirmed by a rheumatologist and dermatologist respectively.

Blood samples were obtained by venipuncture for all subjects, and genomic DNA was isolated from whole blood by standard procedures.

Replication cohorts were from both the U.K. and the U.S. The U.K. cohort consisted of 576 PSA patients from the UK and are described elsewhere [60]. In brief, PSA patients under active follow up by hospital rheumatologists were recruited from throughout the UK although the majority came from the North-West region of England. All patients satisfied the inclusion criteria of having both clinically documented inflammatory synovitis and PS. Each patient was assessed by a trained research nurse, who undertook a standardized clinical history and examination. Detailed demographic and clinical information was obtained and whole blood was taken for DNA extraction and subsequent genetic analysis. Control samples (n = 480) were obtained from blood donors. All patients and controls were white and of UK descent. They were recruited with ethical committee approval (MREC 99/8/84) and provided written informed consent.

The replication cohort from the U.S. for cases consisted of 577 patients with PS (94 of these were also diagnosed with PSA), ascertained at the University of California, San Francisco, CA or at Texas Dermatology, Dallas, TX The replication cohort for controls consisted of 479 unrelated Caucasian individuals from the University of California, San Francisco, ascertained as a set of healthy controls, for cardiovascular studies. A separate cohort of 258 controls was ascertained in Texas. The latter controls were all >40 years of age and were ascertained on the basis of not having PS, PSA, or any other inflammatory or autoimmune disease.

Table S1 also provides information on how well the cases and controls were matched in terms of age and gender. It can be seen that the gender proportions and ages are similar in cases and controls, for both discovery and replication studies.

### Genotyping Methods

DNA was normalized to a concentration of 100 ng/µl (diluted in 10 mM Tris/1 mM EDTA). Samples were quantitated with a Nanodrop Spectrophotometer (ND-1000). For the discovery phase, approximately 1 µg of genomic DNA was used to genotype each sample on the Illumina HumanHap300v2A Genotyping BeadChip. This was performed at the Robert S. Boas Center for Genomics and Human Genetics at The Feinstein Institute for Medical Research, Manhasset, NY. This assay relies on allele specific primer extension and the use of a single fluorochrome. Samples were processed according to the standard Illumina Infinium II automated protocol. This involved whole genome amplification, fragmentation, precipitation, resuspension in hybridization buffer and hybridization to the Illumina Bead Chips for a minimum of 16 h at 48°C. After hybridization the BeadChips were processed for the single base extension reaction, followed by staining and imaging on an Illumina Bead Array Reader. Normalized bead intensity data were loaded into the Illumina Beadstudio 2.0 software which converted fluorescence intensities into SNP genotypes.

Genotyping for all the replication studies was performed with the Sequenom MassArray system (iPlex assay). This involves primer extension chemistry and mass spectrometric analysis described at our web site http://hg.wustl.edu/info/Sequenom_description.html.

### Quality Control

Before analysis, we performed quality filtering of both samples and SNPs to ensure robust association tests. Based on previous criteria [61], we required that all samples used for the discovery phase pass a 93% genotyping call rate threshold, and that all SNPs pass a 95% call rate threshold.

In the case of the replication studies, 57 individuals from the total of 2370 individuals in the replication study were removed because of low genotyping (i.e. when over half of the genotypes for a sample were missing). SNPs with <75% call rates were also excluded from analysis to obtain an average genotyping rate of 0.902. Genotypes were also evaluated for departure from HWE in the controls and SNPs with P<0.001 were removed from further analysis. After pruning, 244 SNPs remained.

A total of 463 ancestry informative SNPs (AIM) present on the Illumina HumanHap300v2A Genotype BeadChip were used to check for possible confounding population substructure in the discovery sample with STRUCTURE software [[62]. For this analysis, genotypes at these SNPs were analyzed for all 742 samples (223 PS cases and 519 controls).

To investigate other biases[20] that could be introduced with shared controls we assessed the potential effect of substructure with the genomic-control method [21] and with EIGENSTRAT [23].

### Statistical Analysis for Association

The Cochran-Armitage Test for trend[25] was conducted with Purcell's PLINK program (http://pngu.mgh.harvard.edu/purcell/plink). However, several SNPs in the current study that exhibited significant differences in cases/controls, were also different when allele frequencies in controls were compared with those from European CEPH typed for SNPs in the HapMap project. NYCP participants are quite diverse with respect to European origin, and many SNPs are reported to show differences among European subgroups[63]. These were identified by a comparison of SNP allele frequencies in European CEPH individuals typed for the HapMap project and were not selected for follow-up studies.

Measures of linkage disequilibrium, D' and r^2^, and allele frequencies were based on precomputed scores from the International HapMap website or were computed locally from HapMap genotypes or from our own case and control genotypes with Haploview 3.2 (http://www.broad.mit.edu/mpg/haploview/). Power calculations for association were calculated at: http://pngu.mgh.harvard.edu/purcell/gpc/. Association localization plots (Figures 2–4,6,S4–S5) were generated with an R code modified from snp.plotter (http://cbdb.nimh.nih.gov/kristin/snp.plotter.html) and Regional Association Plot (http://www.broad.mit.edu/diabetes/scandinavs/figures.html).

### Family Based Tests

Family based association tests on 271 nuclear families were performed with the Pedigree Transmission Disequibrium Test [64] as described elsewhere [11],[12],[65].

### Immunohistochemistry

Tissue sections were fixed with acetone and stained with 10 ug/mL purified mouse anti-human monoclonal antibodies to IL-2 (R&D, clone 5334.21), IL-21 (R&D, J148-1134), COG6 (Abnova, H00057511-M01) and SPPL2A (Abgent, AP6312a). Biotin labeled horse anti-mouse antibodies (Vector Laboratories) were amplified with avidin-biotin complex (Vector Laboratories) and developed with chromogen 3-amino-9-ethylcarbazole (Sigma Aldrich).

## Supporting Information

Table S1 Summary of cases and controls used in discovery and replication stages.
(0.03 MB DOC)

Table S2 Top ranking SNPs where P<5×10^−5^ in discovery cohorts (also stratified on the basis of the presence of PSA or no PSA).
(0.25 MB PDF)

Figure S1 Heterozygosity of sample versus genotyping call rate.
(0.95 MB TIF)

Figure S2 Distribution of SNP success rate in the discovery study.
(1.36 MB TIF)

Figure S3 Q-Q plot of GWA analyses in unrelated individuals used in the discovery study obtained with PLINK. Black dots indicate the negative log of unadjusted trend P values (λ_GC_ = 1.101) and red dots indicate the negative logarithm of adjusted trend P values (λ_GC_ = 1).
(6.03 MB TIF)

Figure S4 Association localization plot for replicated region at chromosome 1q21. Results for SNPs used in the discovery phase (adjusted for GC) are presented as diamonds. Negative LOG P values are provided on the Y axis. The X axis corresponds to the locations of SNPs. The P value for all samples (original GWA scan plus replication samples) are shown as circles. The P value obtained with the most highly associated SNP (from the original GWA scan plus the replication samples) is shown as a red circle. The SNPs shown as orange diamonds are in r2>0.8 (European HapMap CEPH (CEU) samples) with the most significant SNP identified in our study. The recombination rate based on the CEU HapMap is shown in light blue along the x axis (scale on the right). The green arrows indicate the locations of select genes. The LD relationship of Illumina discovery SNPs derived from CEU HapMap genotypes are shown below the graph. The most highly associated SNP, rs6701216 is indicated with an asterisk above the LD plot.
(9.70 MB TIF)

Figure S5 Association localization plots for autoimmune locus on chromosome 4q27 showing P values obtained in discovery sample. Symbols are the same as those used in Figure S4. The asterisk above the LD plot corresponds to rs6840978.
(9.59 MB TIF)
