# Supplementary material for: A Genome-Wide Association Study of Psoriasis and Psoriatic Arthritis Identifies New Disease Loci
Source: PLoS Genet. 2008 Apr 4;4(4):e1000041. doi: 10.1371/journal.pgen.1000041 (PMC2274885; doi:10.1371/journal.pgen.1000041)
Supplement: Table S1 — Summary of cases and controls used in discovery and replication stages. (0.03 MB DOC) [file pgen.1000041.s001.doc]

Table S1. Summary of cases and controls used in discovery and replication stages

|  |  | Discovery Study  (n = 742) | | Replication Study  (n = 2370) | |
| --- | --- | --- | --- | --- | --- |
|  |  | Cases | Control | Cases | Control |
| n |  | 223 | 519 | 1153 | 1217 |
| Sex |  |  |  |  |  |
|  | Males | 46.40% | 44.51% | 45.93% | 48.64% |
|  | Females | 53.64% | 55.49% | 54.07% | 51.36% |
| Age (years) |  |  |  |  |  |
|  | Mean ± SD | 49.8 ± 14.6 | 46.0 ± 11.9 | 44.2 ± 16.4 | 56.5 ± 15.7 |
|  | Range | 12-92 | 21-81 | 2-94 | 20-94 |
| Site |  |  |  |  |  |
|  | USA | 223 | 519 | 577 | 737 |
|  | UK | NA | NA | 576 | 480 |
