# Supplementary material for: A Genome-Wide Association Study of Psoriasis and Psoriatic Arthritis Identifies New Disease Loci
Source: PLoS Genet. 2008 Apr 4;4(4):e1000041. doi: 10.1371/journal.pgen.1000041 (PMC2274885; doi:10.1371/journal.pgen.1000041)
Supplement: Table S2 — Top ranking SNPs where P<5×10^−5^ in discovery cohorts (also stratified on the basis of the presence of PSA or no PSA). (0.25 MB PDF) [file pgen.1000041.s002.pdf]

Supplementary Table 2A. Top ranking hits in all cases used for discovery. MapInfo is location from hg18. OR (odds ratio with the 95% CI). Minor allele frequencies for cases and controls and Trend P values (unadjusted and adjusted by GC) are also provided.

					All Cases				
RS #	CHR	MapInfo (bp)	Minor Allele	HW P (controls)	OR	MAF(cases)	MAF(controls)	Trend P	Trend P (adj.)
rs880051	1	1483590	A	0.594	1.457 [1.120-1.894]	0.290	0.219	6.023E-03	8.862E-03
rs10914850	1	34268681	G	1.000	1.867 [1.178-2.960]	0.081	0.045	7.704E-03	1.111E-02
rs4653108	1	35100619	A	0.762	1.594 [1.216-2.091]	0.258	0.179	8.118E-04	1.420E-03
rs11207808	1	61919026	A	0.674	0.595 [0.453-0.781]	0.206	0.303	1.852E-04	3.667E-04
rs12130439	1	61943203	A	0.659	0.596 [0.451-0.788]	0.190	0.282	2.854E-04	5.465E-04
rs1506399	1	63946072	C	1.000	2.616 [1.402-4.879]	0.050	0.020	1.946E-03	3.152E-03
rs930145	1	68402118	G	0.705	1.706 [1.269-2.292]	0.211	0.135	3.672E-04	6.873E-04
rs1408668	1	86264437	G	0.289	1.786 [1.368-2.330]	0.282	0.180	2.064E-05	4.961E-05
rs1356394	1	102829858	A	0.711	0.077 [0.019-0.317]	0.005	0.063	4.008E-06	1.114E-05
rs316941	1	104912966	G	0.628	1.777 [1.281-2.464]	0.168	0.102	6.724E-04	1.196E-03
rs12066799	1	109321084	A	0.590	0.617 [0.487-0.782]	0.343	0.458	7.166E-05	1.542E-04
rs1217401	1	114240474	G	0.273	1.761 [1.389-2.233]	0.412	0.285	2.565E-06	7.403E-06
rs971173	1	114249437	A	0.226	1.774 [1.399-2.249]	0.412	0.283	2.038E-06	6.008E-06
rs10494809	1	198398707	A	0.759	1.708 [1.304-2.237]	0.269	0.177	9.179E-05	1.935E-04
rs7520626	1	202488465	C	0.132	0.644 [0.503-0.823]	0.287	0.384	3.490E-04	6.546E-04
rs2802846	1	203249336	A	0.394	1.476 [1.163-1.873]	0.384	0.297	1.462E-03	2.422E-03
rs11800413	1	235069500	G	0.372	1.550 [1.227-1.957]	0.461	0.356	2.908E-04	5.545E-04
rs6428942	1	243885338	G	1.000	1.513 [1.120-2.044]	0.194	0.137	7.267E-03	1.053E-02
rs4246561	2	1142434	G	0.166	2.385 [1.498-3.798]	0.088	0.039	2.061E-04	4.061E-04
rs921322	2	11237111	A	0.046	1.332 [1.060-1.674]	0.510	0.438	1.162E-02	1.619E-02
rs162330	2	38173000	C	0.149	1.701 [1.347-2.147]	0.592	0.461	1.833E-05	4.443E-05
rs13032336	2	67568265	A	0.236	1.765 [1.311-2.376]	0.211	0.131	8.609E-05	1.826E-04
rs1518722	2	87410648	C	0.617	0.053 [0.007-0.389]	0.003	0.047	4.225E-05	9.527E-05
rs6749984	2	124156863	G	1.000	2.702 [1.671-4.369]	0.089	0.035	2.165E-05	5.179E-05
rs1439563	2	188566032	G	0.773	1.885 [1.331-2.669]	0.149	0.085	2.507E-04	4.838E-04
rs13432541	2	233817248	A	0.642	2.067 [1.362-3.137]	0.104	0.053	4.946E-04	8.995E-04
rs2697135	3	11052898	A	0.310	0.293 [0.169-0.508]	0.041	0.126	3.703E-06	1.037E-05
rs12487468	3	51364610	G	1.000	2.968 [1.450-6.078]	0.040	0.014	1.641E-03	2.697E-03
rs1461796	3	55199120	A	0.548	1.712 [1.311-2.237]	0.275	0.181	8.812E-05	1.862E-04
rs4974135	3	56353303	A	1.000	2.340 [1.255-4.365]	0.048	0.021	5.278E-03	7.854E-03
rs2193550	3	70538343	C	0.063	1.744 [1.342-2.266]	0.294	0.193	2.312E-05	5.512E-05
rs1164059	3	110942724	A	0.753	0.596 [0.454-0.782]	0.214	0.313	2.084E-04	4.101E-04

					All Cases				
RS #	CHR	MapInfo (bp)	Minor Allele	HW P (controls)	OR	MAF(cases)	MAF(controls)	Trend P	Trend P (adj.)
rs6779681	3	138884283	G	0.471	1.442 [1.123-1.851]	0.320	0.246	3.503E-03	5.398E-03
rs10513502	3	158184555	A	0.439	2.262 [1.371-3.732]	0.073	0.034	1.174E-03	1.987E-03
rs3792686	4	4443969	G	0.313	1.597 [1.270-2.007]	0.535	0.419	6.670E-05	1.448E-04
rs13125385	4	32113322	A	0.199	1.707 [1.177-2.476]	0.124	0.077	5.248E-03	7.814E-03
rs13151961	4	123334952	G	0.112	0.444 [0.309-0.638]	0.095	0.192	1.622E-05	3.980E-05
rs6822844	4	123728871	A	0.054	0.459 [0.320-0.660]	0.095	0.186	4.253E-05	9.620E-05
rs6840978	4	123774157	A	0.053	0.488 [0.352-0.676]	0.123	0.224	2.850E-05	6.646E-05
rs10519368	4	136925018	A	0.757	2.006 [1.405-2.865]	0.144	0.078	1.190E-04	2.452E-04
rs2612182	4	137918325	A	0.298	0.073 [0.018-0.299]	0.006	0.071	5.836E-06	1.569E-05
rs4296721	4	157328717	A	0.591	2.345 [1.492-3.683]	0.093	0.042	1.412E-04	2.863E-04
rs6835397	4	187930570	A	0.650	0.626 [0.494-0.794]	0.343	0.454	8.683E-05	1.844E-04
rs10062995	5	25448500	A	0.075	1.527 [1.153-2.022]	0.232	0.165	3.069E-03	4.782E-03
rs10036252	5	25487576	G	0.101	1.456 [1.040-2.038]	0.147	0.106	2.601E-02	3.391E-02
rs6883825	5	36460847	A	0.921	1.482 [1.174-1.871]	0.429	0.336	9.384E-04	1.614E-03
rs10515168	5	73242423	A	1.000	1.739 [1.171-2.581]	0.109	0.066	5.846E-03	8.627E-03
rs152599	5	106807204	A	0.023	1.626 [1.288-2.052]	0.464	0.347	8.319E-05	1.774E-04
rs1002916	5	117962940	G	0.536	2.308 [1.445-3.685]	0.085	0.039	4.046E-04	7.505E-04
rs4129371	5	120480755	A	0.280	1.885 [1.385-2.567]	0.198	0.115	8.284E-05	1.765E-04
rs13436259	5	120502430	A	0.353	1.976 [1.446-2.701]	0.194	0.109	2.727E-05	6.395E-05
rs7726945	5	128062298	A	1.000	0.505 [0.366-0.698]	0.129	0.226	2.658E-05	6.244E-05
rs4868753	5	164582872	G	0.503	1.402 [1.113-1.767]	0.452	0.370	3.618E-03	5.560E-03
rs4367315	5	164593117	G	0.923	1.401 [1.111-1.766]	0.453	0.371	4.157E-03	6.313E-03
rs1422680	5	164598962	G	0.389	1.400 [1.112-1.763]	0.453	0.371	3.685E-03	5.653E-03
rs3846949	6	15479222	A	0.757	1.528 [1.208-1.933]	0.415	0.317	3.813E-04	7.112E-04
rs4713380	6	30893252	G	0.057	1.833 [1.384-2.427]	0.246	0.151	2.795E-05	6.551E-05
rs4713385	6	30895572	A	0.057	1.785 [1.344-2.370]	0.241	0.151	7.791E-05	1.666E-04
rs7756521	6	30956232	G	1.000	1.895 [1.461-2.457]	0.308	0.190	1.036E-06	3.245E-06
rs1264323	6	30963886	A	0.781	1.609 [1.280-2.023]	0.519	0.401	3.449E-05	7.937E-05
rs1049623	6	30972808	G	0.781	1.609 [1.280-2.023]	0.519	0.401	3.449E-05	7.937E-05
rs3873332	6	31003969	G	1.000	1.906 [1.441-2.520]	0.256	0.153	4.095E-06	1.135E-05
rs3873334	6	31004126	A	0.749	1.776 [1.352-2.333]	0.263	0.167	3.130E-05	7.245E-05
rs2532924	6	31040661	A	0.783	0.565 [0.442-0.721]	0.291	0.421	5.422E-06	1.468E-05
rs3871466	6	31091662	G	0.430	1.823 [1.354-2.453]	0.213	0.129	8.327E-05	1.774E-04
rs2523898	6	31101512	A	0.417	0.553 [0.435-0.703]	0.336	0.478	6.184E-07	2.031E-06
rs3130955	6	31162490	A	0.211	1.579 [1.254-1.987]	0.476	0.365	5.495E-05	1.212E-04

					All Cases				
RS #	CHR	MapInfo (bp)	Minor Allele	HW P (controls)	OR	MAF(cases)	MAF(controls)	Trend P	Trend P (adj.)
rs2233956	6	31189184	G	0.879	1.739 [1.330-2.275]	0.277	0.181	5.197E-05	1.155E-04
rs3094211	6	31194381	G	0.904	0.509 [0.369-0.703]	0.142	0.245	4.148E-05	9.391E-05
rs3094205	6	31199841	G	0.838	1.584 [1.254-2.001]	0.431	0.324	9.650E-05	2.031E-04
rs3131003	6	31201461	A	0.928	1.658 [1.318-2.085]	0.564	0.438	1.335E-05	3.335E-05
rs3815087	6	31201566	A	0.443	1.946 [1.516-2.499]	0.362	0.225	1.292E-07	4.865E-07
rs746647	6	31222161	G	0.413	1.677 [1.328-2.118]	0.441	0.320	8.885E-06	2.298E-05
rs1265112	6	31225998	G	0.413	1.677 [1.328-2.118]	0.441	0.320	8.885E-06	2.298E-05
rs130065	6	31230479	A	0.295	1.935 [1.501-2.496]	0.355	0.221	6.420E-07	2.100E-06
rs3130453	6	31232828	G	0.284	0.531 [0.420-0.672]	0.358	0.512	6.545E-08	2.627E-07
rs720465	6	31233756	A	0.396	1.949 [1.540-2.466]	0.457	0.301	1.266E-08	5.879E-08
rs1419881	6	31238572	A	0.786	1.576 [1.254-1.982]	0.554	0.441	9.363E-05	1.973E-04
rs1265159	6	31248026	A	0.614	2.075 [1.623-2.654]	0.383	0.230	3.384E-09	1.766E-08
rs3130473	6	31307187	A	0.573	1.943 [1.531-2.465]	0.422	0.273	6.819E-08	2.715E-07
rs3130685	6	31314185	A	0.786	1.582 [1.258-1.990]	0.557	0.443	7.954E-05	1.698E-04
rs2394963	6	31359441	A	0.144	0.619 [0.467-0.821]	0.189	0.273	1.123E-03	1.910E-03
rs3873379	6	31370148	G	0.177	0.633 [0.479-0.838]	0.193	0.274	1.724E-03	2.822E-03
rs2894207	6	31371730	G	0.895	1.903 [1.480-2.446]	0.345	0.217	3.735E-07	1.279E-06
rs9366778	6	31377152	A	0.136	0.572 [0.447-0.734]	0.275	0.398	1.658E-05	4.057E-05
rs10484554	6	31382534	A	0.234	2.509 [1.922-3.275]	0.320	0.158	8.747E-12	7.804E-11
rs2523619	6	31426123	G	0.809	1.688 [1.317-2.162]	0.353	0.244	2.787E-05	6.519E-05
rs2596503	6	31428789	A	1.000	0.446 [0.298-0.667]	0.079	0.162	7.870E-05	1.682E-04
rs2523608	6	31430538	G	0.565	1.706 [1.354-2.150]	0.500	0.369	4.003E-06	1.114E-05
rs7743761	6	31444079	A	0.718	1.738 [1.360-2.222]	0.362	0.246	1.769E-05	4.318E-05
rs2523535	6	31444229	G	0.781	0.582 [0.455-0.745]	0.283	0.404	1.598E-05	3.924E-05
rs2844529	6	31461572	A	0.433	1.733 [1.375-2.183]	0.483	0.351	4.397E-06	1.214E-05
rs2428486	6	31462083	G	0.433	1.750 [1.389-2.204]	0.486	0.351	2.902E-06	8.297E-06
rs13437082	6	31462539	A	0.642	2.208 [1.738-2.806]	0.433	0.257	1.399E-10	9.698E-10
rs4711269	6	31462798	A	0.642	2.257 [1.778-2.866]	0.438	0.257	4.009E-11	3.116E-10
rs2523467	6	31470909	A	0.327	1.698 [1.346-2.142]	0.478	0.351	1.128E-05	2.862E-05
rs1051794	6	31487088	A	0.408	1.715 [1.357-2.168]	0.438	0.313	7.128E-06	1.881E-05
rs1131896	6	31487094	A	0.103	0.536 [0.397-0.724]	0.158	0.259	6.976E-05	1.505E-04
rs7772549	6	31515622	G	0.264	1.702 [1.347-2.151]	0.452	0.326	1.128E-05	2.862E-05
rs9469003	6	31515807	G	0.281	2.030 [1.535-2.685]	0.258	0.146	9.161E-07	2.896E-06
rs2596480	6	31533964	A	0.606	0.321 [0.181-0.569]	0.033	0.097	3.548E-05	8.130E-05
rs2395029	6	31539759	C	1.000	3.190 [2.025-5.023]	0.107	0.036	1.432E-07	5.345E-07

					All Cases				
RS #	CHR	MapInfo (bp)	Minor Allele	HW P (controls)	OR	MAF(cases)	MAF(controls)	Trend P	Trend P (adj.)
rs2248462	6	31554775	A	0.901	1.697 [1.323-2.178]	0.342	0.234	3.048E-05	7.073E-05
rs2516509	6	31557973	G	0.901	1.691 [1.316-2.172]	0.341	0.234	3.919E-05	8.907E-05
rs2844509	6	31618903	G	0.738	1.607 [1.263-2.044]	0.381	0.277	1.323E-04	2.702E-04
rs9368699	6	31910520	G	1.000	2.664 [1.700-4.173]	0.100	0.040	5.103E-06	1.387E-05
rs12198173	6	32134786	A	0.594	2.143 [1.542-2.977]	0.178	0.092	4.064E-06	1.130E-05
rs13199524	6	32174743	A	0.571	2.203 [1.577-3.076]	0.175	0.088	2.767E-06	7.949E-06
rs12153855	6	32182782	G	0.819	1.941 [1.417-2.659]	0.190	0.108	2.812E-05	6.582E-05
rs3096697	6	32242488	A	0.194	1.592 [1.233-2.056]	0.311	0.221	2.691E-04	5.179E-04
rs3134945	6	32254470	A	0.156	1.564 [1.211-2.021]	0.309	0.222	4.744E-04	8.692E-04
rs8192591	6	32293774	A	1.000	2.689 [1.679-4.308]	0.092	0.036	1.183E-05	2.988E-05
rs2395185	6	32541145	A	0.832	1.677 [1.322-2.126]	0.418	0.300	2.156E-05	5.154E-05
rs560788	6	52938820	A	0.523	1.590 [1.265-1.999]	0.535	0.420	6.564E-05	1.428E-04
rs10484710	6	56213713	A	0.110	N.D.	0.000	0.062	2.280E-06	6.669E-06
rs346298	6	80630001	A	0.075	1.444 [1.118-1.866]	0.300	0.229	6.839E-03	9.958E-03
rs7754913	6	132423548	C	0.689	1.814 [1.345-2.447]	0.209	0.127	1.043E-04	2.173E-04
rs12182899	6	148982366	G	0.904	0.582 [0.432-0.784]	0.159	0.245	2.702E-04	5.179E-04
rs6557200	6	150314225	G	0.108	1.782 [1.339-2.372]	0.236	0.147	3.701E-05	8.449E-05
rs4719601	7	2610133	A	0.541	0.602 [0.462-0.784]	0.223	0.323	1.595E-04	3.201E-04
rs10244600	7	16206679	C	0.085	1.603 [1.262-2.035]	0.406	0.299	2.438E-04	4.722E-04
rs1404398	7	16628533	A	0.292	1.609 [1.203-2.152]	0.220	0.149	2.156E-03	3.462E-03
rs156301	7	24190049	A	0.312	0.557 [0.419-0.741]	0.180	0.283	7.097E-05	1.535E-04
rs12535722	7	129259292	A	1.000	0.262 [0.130-0.526]	0.023	0.084	5.457E-05	1.206E-04
rs1484646	8	10065806	A	0.202	0.550 [0.416-0.727]	0.192	0.302	1.329E-05	3.319E-05
rs4150895	8	86292487	A	0.619	N.D.	0.000	0.048	1.295E-05	3.241E-05
rs39519	8	90915209	A	1.000	1.603 [1.274-2.018]	0.483	0.368	4.989E-05	1.111E-04
rs1827656	8	122260569	A	1.000	1.842 [1.203-2.820]	0.095	0.054	4.353E-03	6.586E-03
rs1847547	8	122260683	A	1.000	1.970 [1.272-3.050]	0.093	0.049	2.079E-03	3.350E-03
rs4733741	8	130857730	A	0.701	0.604 [0.469-0.779]	0.258	0.365	9.581E-05	2.011E-04
rs7024449	9	691026	A	0.369	0.737 [0.573-0.949]	0.271	0.336	2.115E-02	2.803E-02
rs4740820	9	5600381	A	1.000	1.365 [1.066-1.747]	0.331	0.266	1.429E-02	1.957E-02
rs7042098	9	27815454	A	0.447	1.429 [1.124-1.818]	0.367	0.289	3.798E-03	5.812E-03
rs823918	9	103703117	C	0.069	1.864 [1.385-2.509]	0.216	0.128	6.319E-05	1.380E-04
rs10793992	9	133139159	A	0.194	0.618 [0.484-0.788]	0.300	0.410	7.607E-05	1.634E-04
rs10901365	9	133150374	A	0.263	0.605 [0.472-0.775]	0.289	0.402	4.659E-05	1.044E-04
rs1382580	10	23372821	G	0.358	0.428 [0.281-0.653]	0.066	0.142	4.686E-05	1.049E-04

					All Cases				
RS #	CHR	MapInfo (bp)	Minor Allele	HW P (controls)	OR	MAF(cases)	MAF(controls)	Trend P	Trend P (adj.)
rs384037	11	2869129	G	0.083	0.799 [0.633-1.009]	0.374	0.428	5.684E-02	6.955E-02
rs1365057	11	35066251	A	0.793	1.416 [1.091-1.837]	0.280	0.215	7.885E-03	1.134E-02
rs11605924	11	45829667	A	0.204	0.613 [0.483-0.778]	0.382	0.502	8.836E-05	1.871E-04
rs1447170	11	57632797	G	0.279	2.116 [1.460-3.067]	0.135	0.069	6.890E-05	1.491E-04
rs10736719	11	61756322	G	0.410	1.558 [1.233-1.971]	0.419	0.317	1.835E-04	3.649E-04
rs3850942	11	63364623	G	0.106	2.150 [1.400-3.301]	0.100	0.049	3.669E-04	6.873E-04
rs495366	11	102200318	A	0.477	1.637 [1.281-2.091]	0.355	0.252	7.940E-05	1.698E-04
rs2661969	11	134223427	G	0.895	1.529 [1.180-1.980]	0.293	0.213	1.313E-03	2.204E-03
rs1040092	12	58052436	A	0.823	1.447 [1.044-2.007]	0.156	0.113	2.968E-02	3.826E-02
rs7957738	12	58057308	A	1.000	1.473 [1.070-2.028]	0.167	0.119	1.794E-02	2.411E-02
rs1607784	12	76246319	A	0.456	1.902 [1.373-2.635]	0.174	0.100	8.783E-05	1.862E-04
rs995030	12	87414802	A	0.337	1.733 [1.318-2.279]	0.258	0.167	9.925E-05	2.080E-04
rs1022034	12	87442874	A	0.337	1.744 [1.326-2.294]	0.259	0.167	8.251E-05	1.757E-04
rs3782181	12	87477692	C	0.444	1.771 [1.354-2.316]	0.275	0.176	3.517E-05	8.052E-05
rs3886972	12	92161554	A	0.245	2.902 [1.662-5.068]	0.066	0.024	1.314E-04	2.689E-04
rs10840624	12	120785682	G	0.703	1.620 [1.287-2.039]	0.493	0.375	3.714E-05	8.489E-05
rs2760908	13	26844485	G	0.897	1.372 [1.058-1.779]	0.280	0.221	1.800E-02	2.418E-02
rs12864419	13	28053904	C	0.717	0.602 [0.474-0.766]	0.321	0.439	4.175E-05	9.436E-05
rs1186468	13	38847162	A	0.545	0.577 [0.443-0.753]	0.220	0.329	4.180E-05	9.436E-05
rs4514547	13	39136899	G	0.923	0.586 [0.453-0.758]	0.245	0.357	4.829E-05	1.080E-04
rs4569133	13	39137044	A	0.923	0.579 [0.447-0.749]	0.243	0.357	3.343E-05	7.712E-05
rs7993214	13	39248912	A	0.629	0.586 [0.454-0.756]	0.249	0.361	4.681E-05	1.049E-04
rs9533755	13	43530210	G	0.725	2.095 [1.447-3.034]	0.136	0.070	7.099E-05	1.535E-04
rs288726	13	106292420	A	1.000	1.517 [1.159-1.984]	0.261	0.189	2.678E-03	4.221E-03
rs8018041	14	24520721	G	0.850	1.446 [1.067-1.961]	0.189	0.139	1.935E-02	2.583E-02
rs1958589	14	33914127	G	0.406	1.451 [1.053-2.000]	0.166	0.120	2.018E-02	2.684E-02
rs847506	14	35538760	A	1.000	1.996 [1.391-2.864]	0.140	0.076	9.012E-05	1.907E-04
rs7153045	14	55911652	G	0.097	1.583 [1.256-1.996]	0.464	0.354	5.488E-05	1.212E-04
rs1189046	14	55929403	A	0.464	0.653 [0.513-0.831]	0.324	0.423	4.156E-04	7.691E-04
rs2128673	14	56358349	A	0.271	1.836 [1.345-2.505]	0.195	0.117	9.378E-05	1.973E-04
rs431649	14	67647645	G	0.033	1.606 [1.223-2.108]	0.258	0.178	8.349E-04	1.456E-03
rs372143	14	67657091	G	0.033	1.585 [1.208-2.081]	0.256	0.178	1.146E-03	1.938E-03
rs2588833	14	67669245	A	0.065	1.596 [1.216-2.095]	0.256	0.177	9.368E-04	1.614E-03
rs2588832	14	67672520	C	0.076	1.619 [1.238-2.117]	0.268	0.184	5.492E-04	9.921E-04
rs1742501	14	68398902	G	0.735	1.796 [1.232-2.617]	0.123	0.073	2.808E-03	4.409E-03

					All Cases				
RS #	CHR	MapInfo (bp)	Minor Allele	HW P (controls)	OR	MAF(cases)	MAF(controls)	Trend P	Trend P (adj.)
rs17126387	14	89371067	C	1.000	3.162 [1.980-5.049]	0.102	0.035	6.275E-07	2.051E-06
rs6575501	14	94710082	A	1.000	2.211 [1.443-3.387]	0.102	0.049	1.648E-04	3.296E-04
rs11637031	15	48576417	G	0.737	1.738 [1.315-2.297]	0.246	0.158	8.884E-05	1.880E-04
rs8037553	15	48577525	G	1.000	1.798 [1.357-2.383]	0.244	0.152	4.240E-05	9.573E-05
rs4775912	15	49068271	G	0.856	1.698 [1.272-2.267]	0.223	0.144	3.444E-04	6.482E-04
rs3803369	15	49163121	A	0.855	1.759 [1.319-2.346]	0.227	0.143	1.239E-04	2.549E-04
rs893576	15	49224953	G	1.000	1.603 [1.192-2.154]	0.206	0.139	1.571E-03	2.592E-03
rs4775919	15	49233201	G	1.000	1.649 [1.229-2.214]	0.211	0.139	7.267E-04	1.281E-03
rs8041933	15	49405038	A	0.880	1.378 [1.044-1.818]	0.236	0.183	2.279E-02	3.002E-02
rs288579	16	61135127	G	0.323	1.964 [1.423-2.711]	0.180	0.101	5.633E-05	1.241E-04
rs12447462	16	63038923	G	0.805	1.954 [1.413-2.701]	0.178	0.100	4.686E-05	1.049E-04
rs7193343	16	71586661	A	1.000	0.635 [0.453-0.889]	0.120	0.176	8.140E-03	1.168E-02
rs8059522	16	78014317	G	0.597	1.791 [1.350-2.377]	0.239	0.149	4.274E-05	9.620E-05
rs4782905	16	82813923	A	0.744	0.212 [0.091-0.491]	0.017	0.074	8.336E-05	1.774E-04
rs2240601	17	53106111	A	0.065	1.636 [1.248-2.145]	0.261	0.177	4.077E-04	7.542E-04
rs1941384	18	23988934	A	0.856	1.539 [1.146-2.065]	0.206	0.144	3.959E-03	6.036E-03
rs713042	19	1308082	A	0.228	1.798 [1.377-2.346]	0.285	0.181	1.272E-05	3.194E-05
rs3826942	19	1311575	G	0.532	1.646 [1.281-2.115]	0.331	0.231	9.378E-05	1.973E-04
rs8109578	19	10074154	A	1.000	0.223 [0.116-0.432]	0.026	0.108	2.397E-06	6.960E-06
rs1629174	19	44578058	A	1.000	2.222 [1.421-3.474]	0.092	0.044	3.224E-04	6.083E-04
rs2210455	20	9867383	A	1.000	1.585 [1.205-2.084]	0.254	0.176	9.887E-04	1.696E-03
rs6089151	20	30080496	A	0.711	N.D.	0.000	0.063	2.932E-07	1.029E-06
rs6057638	20	30802993	A	0.342	1.544 [1.169-2.038]	0.239	0.169	1.578E-03	2.603E-03
rs6141829	20	30902870	G	0.393	1.604 [1.205-2.134]	0.225	0.153	7.536E-04	1.326E-03
rs2000291	20	30923196	A	0.582	1.797 [1.348-2.396]	0.230	0.142	3.865E-05	8.780E-05
rs1936307	20	30993189	A	0.245	1.521 [1.189-1.946]	0.360	0.270	9.401E-04	1.622E-03
rs2827771	21	23231640	G	1.000	1.618 [1.187-2.205]	0.188	0.125	1.993E-03	3.222E-03
rs2829505	21	25307597	A	0.848	1.590 [1.263-2.003]	0.486	0.372	8.287E-05	1.765E-04
rs3746887	21	39954610	A	0.786	0.503 [0.357-0.709]	0.116	0.207	9.380E-05	1.973E-04
rs2041629	22	15937144	A	0.370	1.402 [1.065-1.846]	0.239	0.183	1.777E-02	2.391E-02
rs433576	22	16828960	A	0.114	0.591 [0.433-0.807]	0.159	0.242	1.657E-03	2.722E-03
rs390407	22	21588231	G	0.340	0.626 [0.469-0.837]	0.175	0.252	1.427E-03	2.374E-03
rs740234	22	29338745	G	0.869	1.709 [1.295-2.255]	0.249	0.162	1.079E-04	2.248E-04
rs5916793	X	107034204	C	1.000	3.361 [1.422-7.944]	0.040	0.012	3.999E-05	9.080E-05
rs4933045	XY	214201	A	0.392	1.524 [1.198-1.940]	0.397	0.301	6.460E-04	1.150E-03

Supplementary Table 2B. Top ranking hits in stratified subsets of cases with psoriasis but no arthritis. MapInfo is location from hg18. OR (odds ratio with the 95% CI). Minor allele frequencies for cases and controls and Trend P values (unadjusted and adjusted by GC) are also provided.

					Psoriasis				
RS #	CHR	Location (bp)	Minor Allele	HW P (controls)	OR	MAF (cases)	MAF (controls)	Trend P	Trend P (corr.)
rs880051	1	1483590	A	0.594	1.112 [0.799-1.547]	0.238	0.219	5.399E-01	5.527E-01
rs10914850	1	34268681	G	1.000	2.640 [1.612-4.325]	0.110	0.045	8.370E-05	1.396E-04
rs4653108	1	35100619	A	0.762	1.918 [1.401-2.626]	0.295	0.179	4.800E-05	8.241E-05
rs11207808	1	61919026	A	0.674	0.494 [0.349-0.701]	0.177	0.303	7.130E-05	1.196E-04
rs12130439	1	61943203	A	0.659	0.462 [0.320-0.668]	0.153	0.282	3.490E-05	6.121E-05
rs1506399	1	63946072	C	1.000	3.559 [1.836-6.900]	0.067	0.020	8.121E-05	1.355E-04
rs930145	1	68402118	G	0.705	2.061 [1.469-2.891]	0.244	0.135	2.826E-05	5.021E-05
rs1408668	1	86264437	G	0.289	1.799 [1.310-2.469]	0.283	0.180	2.950E-04	4.565E-04
rs1356394	1	102829858	A	0.711	0.064 [0.009-0.464]	0.004	0.063	2.309E-04	3.624E-04
rs316941	1	104912966	G	0.628	1.404 [0.930-2.118]	0.138	0.102	1.118E-01	1.236E-01
rs12066799	1	109321084	A	0.590	0.627 [0.471-0.835]	0.346	0.458	1.369E-03	1.932E-03
rs1217401	1	114240474	G	0.273	1.741 [1.309-2.315]	0.409	0.285	1.449E-04	2.333E-04
rs971173	1	114249437	A	0.226	1.753 [1.318-2.332]	0.409	0.283	1.237E-04	2.008E-04
rs10494809	1	198398707	A	0.759	1.613 [1.165-2.233]	0.258	0.177	3.849E-03	5.126E-03
rs7520626	1	202488465	C	0.132	0.551 [0.404-0.750]	0.256	0.384	8.687E-05	1.445E-04
rs2802846	1	203249336	A	0.394	1.213 [0.905-1.626]	0.339	0.297	1.887E-01	2.031E-01
rs11800413	1	235069500	G	0.372	1.309 [0.986-1.739]	0.420	0.356	6.587E-02	7.488E-02
rs6428942	1	243885338	G	1.000	1.104 [0.748-1.629]	0.150	0.137	6.171E-01	6.282E-01
rs4246561	2	1142434	G	0.166	1.786 [0.993-3.213]	0.067	0.039	5.750E-02	6.585E-02
rs921322	2	11237111	A	0.046	1.042 [0.789-1.376]	0.448	0.438	7.616E-01	7.689E-01
rs162330	2	38173000	C	0.149	1.617 [1.222-2.141]	0.580	0.461	1.285E-03	1.827E-03
rs13032336	2	67568265	A	0.236	1.741 [1.222-2.481]	0.209	0.131	1.216E-03	1.727E-03
rs1518722	2	87410648	C	0.617	N.D.	0.000	0.047	5.753E-04	8.572E-04
rs6749984	2	124156863	G	1.000	2.399 [1.356-4.244]	0.079	0.035	1.932E-03	2.677E-03
rs1439563	2	188566032	G	0.773	2.189 [1.473-3.253]	0.169	0.085	6.199E-05	1.051E-04
rs13432541	2	233817248	A	0.642	1.600 [0.946-2.706]	0.083	0.053	7.624E-02	8.601E-02
rs2697135	3	11052898	A	0.310	0.192 [0.083-0.441]	0.027	0.126	1.118E-05	2.096E-05
rs12487468	3	51364610	G	1.000	2.004 [0.800-5.018]	0.028	0.014	1.270E-01	1.394E-01
rs1461796	3	55199120	A	0.548	1.752 [1.275-2.407]	0.279	0.181	5.676E-04	8.443E-04
rs4974135	3	56353303	A	1.000	1.337 [0.562-3.182]	0.028	0.021	5.046E-01	5.181E-01
rs2193550	3	70538343	C	0.063	1.790 [1.311-2.443]	0.299	0.193	1.651E-04	2.643E-04
rs1164059	3	110942724	A	0.753	0.491 [0.346-0.697]	0.183	0.313	6.415E-05	1.083E-04

					Psoriasis				
RS #	CHR	Location (bp)	Minor Allele	HW P (controls)	OR	MAF (cases)	MAF (controls)	Trend P	Trend P (corr.)
rs6779681	3	138884283	G	0.471	1.770 [1.321-2.373]	0.366	0.246	8.481E-05	1.410E-04
rs10513502	3	158184555	A	0.439	1.539 [0.800-2.961]	0.051	0.034	1.948E-01	2.093E-01
rs3792686	4	4443969	G	0.313	1.728 [1.309-2.280]	0.555	0.419	1.259E-04	2.049E-04
rs13125385	4	32113322	A	0.199	1.217 [0.747-1.983]	0.092	0.077	4.389E-01	4.536E-01
rs13151961	4	123334952	G	0.112	0.364 [0.224-0.590]	0.079	0.192	4.338E-05	7.500E-05
rs6822844	4	123728871	A	0.054	0.416 [0.261-0.663]	0.087	0.186	2.808E-04	4.363E-04
rs6840978	4	123774157	A	0.053	0.413 [0.270-0.632]	0.106	0.224	6.331E-05	1.072E-04
rs10519368	4	136925018	A	0.757	2.285 [1.522-3.431]	0.161	0.078	4.919E-05	8.448E-05
rs2612182	4	137918325	A	0.298	0.118 [0.029-0.486]	0.009	0.071	6.765E-04	9.977E-04
rs4296721	4	157328717	A	0.591	1.657 [0.927-2.963]	0.067	0.042	8.345E-02	9.370E-02
rs6835397	4	187930570	A	0.650	0.558 [0.416-0.749]	0.317	0.454	7.428E-05	1.245E-04
rs10062995	5	25448500	A	0.075	1.207 [0.848-1.718]	0.193	0.165	2.877E-01	3.031E-01
rs10036252	5	25487576	G	0.101	0.964 [0.613-1.516]	0.102	0.106	8.681E-01	8.722E-01
rs6883825	5	36460847	A	0.921	1.824 [1.380-2.410]	0.480	0.336	2.096E-05	3.788E-05
rs10515168	5	73242423	A	1.000	1.215 [0.722-2.044]	0.079	0.066	4.690E-01	4.832E-01
rs152599	5	106807204	A	0.023	1.651 [1.246-2.188]	0.468	0.347	7.201E-04	1.055E-03
rs1002916	5	117962940	G	0.536	1.775 [0.987-3.192]	0.067	0.039	5.586E-02	6.407E-02
rs4129371	5	120480755	A	0.280	1.693 [1.165-2.459]	0.181	0.115	7.294E-03	9.368E-03
rs13436259	5	120502430	A	0.353	1.764 [1.208-2.576]	0.177	0.109	4.334E-03	5.734E-03
rs7726945	5	128062298	A	1.000	0.590 [0.403-0.862]	0.147	0.226	5.914E-03	7.687E-03
rs4868753	5	164582872	G	0.503	1.122 [0.846-1.487]	0.398	0.370	4.158E-01	4.307E-01
rs4367315	5	164593117	G	0.923	1.119 [0.843-1.484]	0.398	0.371	4.332E-01	4.478E-01
rs1422680	5	164598962	G	0.389	1.136 [0.858-1.506]	0.402	0.371	3.626E-01	3.780E-01
rs3846949	6	15479222	A	0.757	1.757 [1.327-2.326]	0.449	0.317	7.404E-05	1.245E-04
rs4713380	6	30893252	G	0.057	1.772 [1.266-2.478]	0.240	0.151	1.042E-03	1.498E-03
rs4713385	6	30895572	A	0.057	1.690 [1.200-2.382]	0.232	0.151	3.350E-03	4.498E-03
rs7756521	6	30956232	G	1.000	1.886 [1.384-2.571]	0.307	0.190	5.483E-05	9.376E-05
rs1264323	6	30963886	A	0.781	1.515 [1.149-1.997]	0.504	0.401	2.559E-03	3.489E-03
rs1049623	6	30972808	G	0.781	1.515 [1.149-1.997]	0.504	0.401	2.559E-03	3.489E-03
rs3873332	6	31003969	G	1.000	1.905 [1.369-2.651]	0.256	0.153	1.227E-04	1.998E-04
rs3873334	6	31004126	A	0.749	1.747 [1.261-2.420]	0.260	0.167	8.718E-04	1.266E-03
rs2532924	6	31040661	A	0.783	0.564 [0.419-0.760]	0.291	0.421	2.005E-04	3.165E-04
rs3871466	6	31091662	G	0.430	1.689 [1.181-2.416]	0.201	0.129	4.284E-03	5.672E-03
rs2523898	6	31101512	A	0.417	0.560 [0.418-0.752]	0.339	0.478	7.093E-05	1.190E-04
rs3130955	6	31162490	A	0.211	1.604 [1.215-2.118]	0.480	0.365	4.948E-04	7.403E-04

					Psoriasis				
**RS #**	**CHR**	**Location (bp)**	**Minor Allele**	**HW P (controls)**	**OR**	**MAF (cases)**	**MAF (controls)**	**Trend P**	**Trend P (corr.)**
rs2233956	6	31189184	G	0.879	1.658 [1.201-2.288]	0.268	0.181	1.940E-03	2.688E-03
rs3094211	6	31194381	G	0.904	0.557 [0.378-0.822]	0.153	0.245	3.239E-03	4.356E-03
rs3094205	6	31199841	G	0.838	1.648 [1.244-2.182]	0.441	0.324	4.366E-04	6.592E-04
rs3131003	6	31201461	A	0.928	1.651 [1.251-2.180]	0.563	0.438	3.670E-04	5.611E-04
rs3815087	6	31201566	A	0.443	1.918 [1.426-2.580]	0.358	0.225	1.156E-05	2.170E-05
rs746647	6	31222161	G	0.413	1.625 [1.227-2.154]	0.433	0.320	5.150E-04	7.709E-04
rs1265112	6	31225998	G	0.413	1.625 [1.227-2.154]	0.433	0.320	5.150E-04	7.709E-04
rs130065	6	31230479	A	0.295	1.813 [1.336-2.459]	0.340	0.221	1.620E-04	2.591E-04
rs3130453	6	31232828	G	0.284	0.560 [0.422-0.743]	0.370	0.512	3.559E-05	6.243E-05
rs720465	6	31233756	A	0.396	1.818 [1.367-2.416]	0.439	0.301	2.008E-05	3.642E-05
rs1419881	6	31238572	A	0.786	1.531 [1.161-2.019]	0.547	0.441	2.192E-03	3.016E-03
rs1265159	6	31248026	A	0.614	1.915 [1.424-2.576]	0.364	0.230	1.088E-05	2.045E-05
rs3130473	6	31307187	A	0.573	1.729 [1.297-2.305]	0.394	0.273	1.976E-04	3.134E-04
rs3130685	6	31314185	A	0.786	1.594 [1.208-2.104]	0.559	0.443	7.510E-04	1.099E-03
rs2394963	6	31359441	A	0.144	0.812 [0.588-1.122]	0.234	0.273	2.187E-01	2.336E-01
rs3873379	6	31370148	G	0.177	0.828 [0.601-1.142]	0.238	0.274	2.624E-01	2.778E-01
rs2894207	6	31371730	G	0.895	1.903 [1.409-2.568]	0.345	0.217	2.222E-05	4.000E-05
rs9366778	6	31377152	A	0.136	0.682 [0.508-0.915]	0.311	0.398	1.251E-02	1.558E-02
rs10484554	6	31382534	A	0.234	2.497 [1.823-3.420]	0.319	0.158	1.041E-08	2.976E-08
rs2523619	6	31426123	G	0.809	1.725 [1.281-2.324]	0.358	0.244	2.940E-04	4.542E-04
rs2596503	6	31428789	A	1.000	0.469 [0.288-0.764]	0.083	0.162	2.135E-03	2.941E-03
rs2523608	6	31430538	G	0.565	1.846 [1.399-2.436]	0.520	0.369	1.001E-05	1.890E-05
rs7743761	6	31444079	A	0.718	1.762 [1.313-2.364]	0.365	0.246	2.157E-04	3.395E-04
rs2523535	6	31444229	G	0.781	0.517 [0.380-0.703]	0.260	0.404	2.197E-05	3.961E-05
rs2844529	6	31461572	A	0.433	1.852 [1.403-2.446]	0.500	0.351	1.728E-05	3.156E-05
rs2428486	6	31462083	G	0.433	1.852 [1.403-2.446]	0.500	0.351	1.728E-05	3.156E-05
rs13437082	6	31462539	A	0.642	2.313 [1.738-3.079]	0.444	0.257	1.080E-08	3.079E-08
rs4711269	6	31462798	A	0.642	2.354 [1.771-3.131]	0.449	0.257	4.832E-09	1.442E-08
rs2523467	6	31470909	A	0.327	1.823 [1.378-2.411]	0.496	0.351	3.375E-05	5.942E-05
rs1051794	6	31487088	A	0.408	1.789 [1.351-2.370]	0.449	0.313	5.585E-05	9.517E-05
rs1131896	6	31487094	A	0.103	0.656 [0.464-0.928]	0.186	0.259	1.937E-02	2.354E-02
rs7772549	6	31515622	G	0.264	1.789 [1.351-2.368]	0.464	0.326	6.213E-05	1.051E-04
rs9469003	6	31515807	G	0.281	2.131 [1.535-2.960]	0.268	0.146	7.698E-06	1.478E-05
rs2596480	6	31533964	A	0.606	0.344 [0.171-0.690]	0.035	0.097	1.451E-03	2.044E-03
rs2395029	6	31539759	C	1.000	3.444 [2.068-5.736]	0.114	0.036	6.232E-07	1.389E-06

					Psoriasis				
RS #	CHR	Location (bp)	Minor Allele	HW P (controls)	OR	MAF (cases)	MAF (controls)	Trend P	Trend P (corr.)
rs2248462	6	31554775	A	0.901	1.782 [1.324-2.398]	0.353	0.234	1.293E-04	2.100E-04
rs2516509	6	31557973	G	0.901	1.778 [1.320-2.395]	0.352	0.234	1.495E-04	2.404E-04
rs2844509	6	31618903	G	0.738	1.805 [1.355-2.405]	0.409	0.277	5.771E-05	9.805E-05
rs9368699	6	31910520	G	1.000	3.106 [1.885-5.119]	0.114	0.040	1.674E-06	3.513E-06
rs12198173	6	32134786	A	0.594	2.251 [1.535-3.299]	0.185	0.092	2.326E-05	4.182E-05
rs13199524	6	32174743	A	0.571	2.363 [1.608-3.473]	0.185	0.088	8.500E-06	1.623E-05
rs12153855	6	32182782	G	0.819	1.983 [1.370-2.871]	0.193	0.108	2.414E-04	3.772E-04
rs3096697	6	32242488	A	0.194	1.357 [0.991-1.857]	0.278	0.221	4.877E-02	5.635E-02
rs3134945	6	32254470	A	0.156	1.321 [0.962-1.812]	0.274	0.222	7.495E-02	8.462E-02
rs8192591	6	32293774	A	1.000	2.851 [1.667-4.876]	0.097	0.036	4.429E-05	7.650E-05
rs2395185	6	32541145	A	0.832	1.772 [1.332-2.357]	0.431	0.300	9.716E-05	1.604E-04
rs560788	6	52938820	A	0.523	1.516 [1.150-1.998]	0.524	0.420	2.652E-03	3.610E-03
rs10484710	6	56213713	A	0.110	N.D.	0.000	0.062	2.602E-04	4.047E-04
rs346298	6	80630001	A	0.075	1.076 [0.778-1.488]	0.242	0.229	6.671E-01	6.770E-01
rs7754913	6	132423548	C	0.689	1.569 [1.087-2.265]	0.186	0.127	1.683E-02	2.062E-02
rs12182899	6	148982366	G	0.904	0.428 [0.287-0.641]	0.122	0.245	2.092E-05	3.788E-05
rs6557200	6	150314225	G	0.108	1.670 [1.183-2.357]	0.224	0.147	2.543E-03	3.468E-03
rs4719601	7	2610133	A	0.541	0.752 [0.552-1.024]	0.264	0.323	6.699E-02	7.610E-02
rs10244600	7	16206679	C	0.085	1.844 [1.387-2.451]	0.440	0.299	5.177E-05	8.878E-05
rs1404398	7	16628533	A	0.292	1.198 [0.826-1.738]	0.173	0.149	3.612E-01	3.766E-01
rs156301	7	24190049	A	0.312	0.591 [0.419-0.834]	0.189	0.283	3.099E-03	4.180E-03
rs12535722	7	129259292	A	1.000	0.421 [0.209-0.850]	0.037	0.084	1.254E-02	1.562E-02
rs1484646	8	10065806	A	0.202	0.516 [0.365-0.730]	0.182	0.302	1.033E-04	1.695E-04
rs4150895	8	86292487	A	0.619	N.D.	0.000	0.048	7.293E-04	1.071E-03
rs39519	8	90915209	A	1.000	1.559 [1.181-2.058]	0.476	0.368	1.539E-03	2.162E-03
rs1827656	8	122260569	A	1.000	1.262 [0.718-2.217]	0.067	0.054	4.174E-01	4.322E-01
rs1847547	8	122260683	A	1.000	1.380 [0.781-2.440]	0.067	0.049	2.675E-01	2.830E-01
rs4733741	8	130857730	A	0.701	0.660 [0.487-0.895]	0.276	0.365	7.583E-03	9.719E-03
rs7024449	9	691026	A	0.369	1.013 [0.757-1.355]	0.339	0.336	9.312E-01	9.333E-01
rs4740820	9	5600381	A	1.000	1.785 [1.337-2.384]	0.393	0.266	9.730E-05	1.604E-04
rs7042098	9	27815454	A	0.447	1.763 [1.327-2.343]	0.417	0.289	8.032E-05	1.341E-04
rs823918	9	103703117	C	0.069	2.052 [1.453-2.898]	0.232	0.128	6.586E-05	1.110E-04
rs10793992	9	133139159	A	0.194	0.577 [0.427-0.779]	0.286	0.410	2.341E-04	3.660E-04
rs10901365	9	133150374	A	0.263	0.591 [0.436-0.799]	0.284	0.402	4.688E-04	7.039E-04
rs1382580	10	23372821	G	0.358	0.571 [0.356-0.915]	0.087	0.142	1.631E-02	2.002E-02

					Psoriasis				
RS #	CHR	Location (bp)	Minor Allele	HW P (controls)	OR	MAF (cases)	MAF (controls)	Trend P	Trend P (corr.)
rs384037	11	2869129	G	0.083	1.087 [0.824-1.434]	0.449	0.428	5.464E-01	5.591E-01
rs1365057	11	35066251	A	0.793	1.835 [1.358-2.479]	0.335	0.215	6.178E-05	1.046E-04
rs11605924	11	45829667	A	0.204	0.647 [0.485-0.864]	0.395	0.502	4.067E-03	5.399E-03
rs1447170	11	57632797	G	0.279	2.023 [1.303-3.142]	0.130	0.069	1.746E-03	2.434E-03
rs10736719	11	61756322	G	0.410	1.729 [1.305-2.290]	0.445	0.317	9.714E-05	1.604E-04
rs3850942	11	63364623	G	0.106	1.572 [0.909-2.722]	0.075	0.049	1.094E-01	1.211E-01
rs495366	11	102200318	A	0.477	1.715 [1.280-2.297]	0.366	0.252	3.120E-04	4.800E-04
rs2661969	11	134223427	G	0.895	1.813 [1.339-2.455]	0.329	0.213	8.905E-05	1.474E-04
rs1040092	12	58052436	A	0.823	0.967 [0.624-1.499]	0.110	0.113	8.852E-01	8.888E-01
rs7957738	12	58057308	A	1.000	0.987 [0.644-1.511]	0.118	0.119	9.513E-01	9.528E-01
rs1607784	12	76246319	A	0.456	1.965 [1.340-2.882]	0.179	0.100	3.920E-04	5.960E-04
rs995030	12	87414802	A	0.337	1.641 [1.181-2.282]	0.248	0.167	3.792E-03	5.054E-03
rs1022034	12	87442874	A	0.337	1.624 [1.166-2.261]	0.246	0.167	4.878E-03	6.410E-03
rs3782181	12	87477692	C	0.444	1.607 [1.161-2.224]	0.256	0.176	4.766E-03	6.271E-03
rs3886972	12	92161554	A	0.245	3.872 [2.134-7.027]	0.087	0.024	3.531E-06	7.111E-06
rs10840624	12	120785682	G	0.703	1.516 [1.149-2.001]	0.476	0.375	2.963E-03	4.006E-03
rs2760908	13	26844485	G	0.897	1.023 [0.735-1.423]	0.224	0.221	8.965E-01	8.997E-01
rs12864419	13	28053904	C	0.717	0.558 [0.414-0.750]	0.304	0.439	1.211E-04	1.968E-04
rs1186468	13	38847162	A	0.545	0.539 [0.387-0.749]	0.209	0.329	1.796E-04	2.864E-04
rs4514547	13	39136899	G	0.923	0.558 [0.407-0.766]	0.236	0.357	2.826E-04	4.385E-04
rs4569133	13	39137044	A	0.923	0.552 [0.401-0.759]	0.234	0.357	2.332E-04	3.660E-04
rs7993214	13	39248912	A	0.629	0.559 [0.408-0.766]	0.240	0.361	3.031E-04	4.681E-04
rs9533755	13	43530210	G	0.725	2.369 [1.554-3.613]	0.151	0.070	4.320E-05	7.463E-05
rs288726	13	106292420	A	1.000	1.189 [0.848-1.667]	0.216	0.189	3.208E-01	3.363E-01
rs8018041	14	24520721	G	0.850	1.982 [1.410-2.786]	0.242	0.139	9.972E-05	1.645E-04
rs1958589	14	33914127	G	0.406	1.990 [1.394-2.841]	0.214	0.120	9.608E-05	1.589E-04
rs847506	14	35538760	A	1.000	2.082 [1.368-3.168]	0.146	0.076	3.755E-04	5.725E-04
rs7153045	14	55911652	G	0.097	1.465 [1.108-1.937]	0.445	0.354	5.331E-03	6.972E-03
rs1189046	14	55929403	A	0.464	0.788 [0.590-1.051]	0.366	0.423	9.528E-02	1.062E-01
rs2128673	14	56358349	A	0.271	1.873 [1.298-2.702]	0.198	0.117	6.940E-04	1.018E-03
rs431649	14	67647645	G	0.033	1.975 [1.441-2.707]	0.300	0.178	2.952E-05	5.224E-05
rs372143	14	67657091	G	0.033	1.931 [1.410-2.644]	0.295	0.178	5.257E-05	9.011E-05
rs2588833	14	67669245	A	0.065	1.944 [1.420-2.663]	0.295	0.177	4.141E-05	7.173E-05
rs2588832	14	67672520	C	0.076	1.926 [1.410-2.630]	0.303	0.184	4.566E-05	7.881E-05
rs1742501	14	68398902	G	0.735	0.975 [0.570-1.666]	0.071	0.073	9.265E-01	9.288E-01

					Psoriasis				
RS #	CHR	Location (bp)	Minor Allele	HW P (controls)	OR	MAF (cases)	MAF (controls)	Trend P	Trend P (corr.)
rs17126387	14	89371067	C	1.000	3.216 [1.891-5.469]	0.104	0.035	7.426E-06	1.428E-05
rs6575501	14	94710082	A	1.000	2.709 [1.689-4.347]	0.122	0.049	1.672E-05	3.064E-05
rs11637031	15	48576417	G	0.737	1.644 [1.175-2.299]	0.236	0.158	3.452E-03	4.626E-03
rs8037553	15	48577525	G	1.000	1.685 [1.201-2.365]	0.232	0.152	2.536E-03	3.459E-03
rs4775912	15	49068271	G	0.856	1.345 [0.936-1.932]	0.185	0.144	1.107E-01	1.224E-01
rs3803369	15	49163121	A	0.855	1.428 [0.998-2.042]	0.193	0.143	5.227E-02	6.017E-02
rs893576	15	49224953	G	1.000	1.188 [0.813-1.735]	0.161	0.139	3.695E-01	3.848E-01
rs4775919	15	49233201	G	1.000	1.223 [0.839-1.781]	0.165	0.139	2.906E-01	3.061E-01
rs8041933	15	49405038	A	0.880	0.997 [0.697-1.427]	0.182	0.183	9.886E-01	9.889E-01
rs288579	16	61135127	G	0.323	1.721 [1.162-2.548]	0.161	0.101	7.053E-03	9.077E-03
rs12447462	16	63038923	G	0.805	1.999 [1.367-2.924]	0.181	0.100	3.084E-04	4.752E-04
rs7193343	16	71586661	A	1.000	0.899 [0.620-1.304]	0.161	0.176	5.760E-01	5.881E-01
rs8059522	16	78014317	G	0.597	1.799 [1.286-2.518]	0.240	0.149	4.435E-04	6.693E-04
rs4782905	16	82813923	A	0.744	0.057 [0.008-0.415]	0.005	0.074	1.252E-04	2.038E-04
rs2240601	17	53106111	A	0.065	2.019 [1.477-2.760]	0.303	0.177	1.248E-05	2.325E-05
rs1941384	18	23988934	A	0.856	1.140 [0.782-1.664]	0.161	0.144	4.906E-01	5.044E-01
rs713042	19	1308082	A	0.228	1.822 [1.329-2.498]	0.287	0.181	1.487E-04	2.392E-04
rs3826942	19	1311575	G	0.532	1.576 [1.165-2.133]	0.321	0.231	3.030E-03	4.092E-03
rs8109578	19	10074154	A	1.000	0.305 [0.146-0.634]	0.036	0.108	1.053E-03	1.513E-03
rs1629174	19	44578058	A	1.000	2.703 [1.647-4.437]	0.110	0.044	4.617E-05	7.960E-05
rs2210455	20	9867383	A	1.000	1.289 [0.917-1.813]	0.216	0.176	1.409E-01	1.539E-01
rs6089151	20	30080496	A	0.711	N.D.	0.000	0.063	6.250E-05	1.056E-04
rs6057638	20	30802993	A	0.342	1.866 [1.354-2.572]	0.276	0.169	5.882E-05	1.000E-04
rs6141829	20	30902870	G	0.393	2.018 [1.456-2.797]	0.268	0.153	8.231E-06	1.576E-05
rs2000291	20	30923196	A	0.582	2.336 [1.686-3.237]	0.279	0.142	8.588E-08	2.157E-07
rs1936307	20	30993189	A	0.245	1.870 [1.401-2.497]	0.409	0.270	2.306E-05	4.141E-05
rs2827771	21	23231640	G	1.000	1.267 [0.857-1.872]	0.153	0.125	2.256E-01	2.405E-01
rs2829505	21	25307597	A	0.848	1.530 [1.157-2.023]	0.476	0.372	2.820E-03	3.824E-03
rs3746887	21	39954610	A	0.786	0.504 [0.328-0.774]	0.116	0.207	1.639E-03	2.292E-03
rs2041629	22	15937144	A	0.370	1.038 [0.730-1.477]	0.189	0.183	8.386E-01	8.436E-01
rs433576	22	16828960	A	0.114	0.417 [0.272-0.638]	0.117	0.242	8.976E-05	1.489E-04
rs390407	22	21588231	G	0.340	0.450 [0.304-0.667]	0.132	0.252	4.475E-05	7.726E-05
rs740234	22	29338745	G	0.869	2.041 [1.482-2.812]	0.283	0.162	7.816E-06	1.500E-05
rs5916793	X	107034204	C	1.000	3.120 [1.146-8.489]	0.037	0.012	3.735E-05	6.528E-05
rs4933045	XY	214201	A	0.392	1.786 [1.341-2.378]	0.435	0.301	8.527E-05	1.417E-04

Supplementary Table 2C. Top ranking hits in stratified subsets of cases with only psoriatic arthritis. MapInfo is location from hg18. OR (odds ratio with the 95% CI). Minor allele frequencies for cases and controls and Trend P values (unadjusted and adjusted by GC) are also provided.

					Psoriatic Arthritis				
RS #	CHR	MapInfo (bp)	Minor Allele	HW P (controls)	OR	MAF(cases)	MAF(controls)	Trend P	Trend P (adj.)
rs880051	1	1483590	A	0.594	2.095 [1.471-2.984]	0.370	0.219	3.937E-05	6.221E-05
rs10914850	1	34268681	G	1.000	0.789 [0.331-1.880]	0.036	0.045	5.915E-01	6.012E-01
rs4653108	1	35100619	A	0.762	1.162 [0.771-1.749]	0.202	0.179	4.769E-01	4.884E-01
rs11207808	1	61919026	A	0.674	0.765 [0.524-1.117]	0.250	0.303	1.666E-01	1.779E-01
rs12130439	1	61943203	A	0.659	0.823 [0.564-1.200]	0.244	0.282	3.128E-01	3.255E-01
rs1506399	1	63946072	C	1.000	1.230 [0.415-3.645]	0.024	0.020	7.054E-01	7.126E-01
rs930145	1	68402118	G	0.705	1.222 [0.780-1.916]	0.161	0.135	3.767E-01	3.892E-01
rs1408668	1	86264437	G	0.289	1.766 [1.216-2.565]	0.280	0.180	3.136E-03	4.009E-03
rs1356394	1	102829858	A	0.711	0.097 [0.013-0.708]	0.006	0.063	3.908E-03	4.945E-03
rs316941	1	104912966	G	0.628	2.396 [1.572-3.652]	0.214	0.102	4.143E-05	6.540E-05
rs12066799	1	109321084	A	0.590	0.602 [0.426-0.850]	0.337	0.458	3.707E-03	4.702E-03
rs1217401	1	114240474	G	0.273	1.793 [1.282-2.509]	0.417	0.285	6.778E-04	9.318E-04
rs971173	1	114249437	A	0.226	1.806 [1.290-2.527]	0.417	0.283	6.005E-04	8.326E-04
rs10494809	1	198398707	A	0.759	1.856 [1.280-2.692]	0.286	0.177	1.056E-03	1.419E-03
rs7520626	1	202488465	C	0.132	0.801 [0.567-1.130]	0.333	0.384	1.968E-01	2.087E-01
rs2802846	1	203249336	A	0.394	1.957 [1.403-2.729]	0.452	0.297	7.735E-05	1.183E-04
rs11800413	1	235069500	G	0.372	1.991 [1.429-2.775]	0.524	0.356	5.959E-05	9.200E-05
rs6428942	1	243885338	G	1.000	2.227 [1.511-3.282]	0.262	0.137	4.639E-05	7.267E-05
rs4246561	2	1142434	G	0.166	3.337 [1.894-5.878]	0.119	0.039	1.346E-05	2.244E-05
rs921322	2	11237111	A	0.046	1.942 [1.389-2.715]	0.602	0.438	5.404E-05	8.405E-05
rs162330	2	38173000	C	0.149	1.840 [1.310-2.585]	0.611	0.461	5.967E-04	8.242E-04
rs13032336	2	67568265	A	0.236	1.801 [1.193-2.718]	0.214	0.131	3.582E-03	4.552E-03
rs1518722	2	87410648	C	0.617	0.139 [0.019-1.012]	0.007	0.047	1.992E-02	2.337E-02
rs6749984	2	124156863	G	1.000	3.174 [1.730-5.827]	0.102	0.035	6.151E-05	9.481E-05
rs1439563	2	188566032	G	0.773	1.452 [0.865-2.434]	0.119	0.085	1.570E-01	1.680E-01
rs13432541	2	233817248	A	0.642	2.816 [1.675-4.736]	0.137	0.053	5.727E-05	8.882E-05
rs2697135	3	11052898	A	0.310	0.454 [0.225-0.913]	0.062	0.126	2.226E-02	2.598E-02
rs12487468	3	51364610	G	1.000	4.476 [1.954-10.250]	0.060	0.014	9.547E-05	1.447E-04
rs1461796	3	55199120	A	0.548	1.652 [1.133-2.410]	0.268	0.181	7.877E-03	9.645E-03
rs4974135	3	56353303	A	1.000	3.926 [1.926-8.002]	0.077	0.021	4.173E-05	6.573E-05
rs2193550	3	70538343	C	0.063	1.677 [1.158-2.427]	0.286	0.193	4.603E-03	5.780E-03
rs1164059	3	110942724	A	0.753	0.767 [0.529-1.114]	0.259	0.313	1.683E-01	1.796E-01

					Psoriatic Arthritis				
RS #	CHR	MapInfo (bp)	Minor Allele	HW P (controls)	OR	MAF(cases)	MAF(controls)	Trend P	Trend P (adj.)
rs6779681	3	138884283	G	0.471	1.022 [0.700-1.490]	0.250	0.246	9.106E-01	9.129E-01
rs10513502	3	158184555	A	0.439	3.424 [1.885-6.217]	0.107	0.034	2.689E-05	4.317E-05
rs3792686	4	4443969	G	0.313	1.418 [1.022-1.968]	0.506	0.419	3.900E-02	4.436E-02
rs13125385	4	32113322	A	0.199	2.506 [1.578-3.981]	0.173	0.077	9.080E-05	1.376E-04
rs13151961	4	123334952	G	0.112	0.570 [0.348-0.933]	0.119	0.192	2.953E-02	3.403E-02
rs6822844	4	123728871	A	0.054	0.527 [0.315-0.880]	0.107	0.186	1.786E-02	2.106E-02
rs6840978	4	123774157	A	0.053	0.607 [0.387-0.952]	0.149	0.224	3.650E-02	4.164E-02
rs10519368	4	136925018	A	0.757	1.604 [0.953-2.701]	0.119	0.078	7.276E-02	8.049E-02
rs2612182	4	137918325	A	0.298	N.D.	0.000	0.071	1.676E-03	2.208E-03
rs4296721	4	157328717	A	0.591	3.451 [2.002-5.949]	0.131	0.042	2.787E-06	5.006E-06
rs6835397	4	187930570	A	0.650	0.739 [0.528-1.033]	0.381	0.454	7.234E-02	8.006E-02
rs10062995	5	25448500	A	0.075	2.079 [1.433-3.015]	0.292	0.165	7.665E-05	1.171E-04
rs10036252	5	25487576	G	0.101	2.305 [1.515-3.508]	0.214	0.106	6.198E-05	9.577E-05
rs6883825	5	36460847	A	0.921	1.068 [0.758-1.505]	0.351	0.336	7.063E-01	7.136E-01
rs10515168	5	73242423	A	1.000	2.602 [1.599-4.235]	0.155	0.066	6.338E-05	9.771E-05
rs152599	5	106807204	A	0.023	1.590 [1.142-2.212]	0.458	0.347	8.966E-03	1.091E-02
rs1002916	5	117962940	G	0.536	3.155 [1.776-5.606]	0.113	0.039	4.791E-05	7.489E-05
rs4129371	5	120480755	A	0.280	2.196 [1.452-3.320]	0.223	0.115	2.012E-04	2.934E-04
rs13436259	5	120502430	A	0.353	2.314 [1.527-3.506]	0.220	0.109	6.697E-05	1.033E-04
rs7726945	5	128062298	A	1.000	0.386 [0.229-0.651]	0.101	0.226	2.354E-04	3.399E-04
rs4868753	5	164582872	G	0.503	1.967 [1.411-2.743]	0.537	0.370	4.633E-05	7.267E-05
rs4367315	5	164593117	G	0.923	1.955 [1.406-2.720]	0.536	0.371	5.999E-05	9.293E-05
rs1422680	5	164598962	G	0.389	1.908 [1.373-2.651]	0.530	0.371	8.467E-05	1.288E-04
rs3846949	6	15479222	A	0.757	1.230 [0.874-1.730]	0.363	0.317	2.375E-01	2.500E-01
rs4713380	6	30893252	G	0.057	1.928 [1.309-2.840]	0.256	0.151	1.063E-03	1.434E-03
rs4713385	6	30895572	A	0.057	1.928 [1.309-2.840]	0.256	0.151	1.063E-03	1.434E-03
rs7756521	6	30956232	G	1.000	1.908 [1.327-2.743]	0.309	0.190	3.560E-04	5.051E-04
rs1264323	6	30963886	A	0.781	1.762 [1.269-2.448]	0.542	0.401	6.516E-04	8.990E-04
rs1049623	6	30972808	G	0.781	1.762 [1.269-2.448]	0.542	0.401	6.516E-04	8.990E-04
rs3873332	6	31003969	G	1.000	1.907 [1.290-2.819]	0.256	0.153	8.194E-04	1.115E-03
rs3873334	6	31004126	A	0.749	1.821 [1.246-2.661]	0.268	0.167	1.522E-03	2.016E-03
rs2532924	6	31040661	A	0.783	0.565 [0.396-0.807]	0.292	0.421	1.547E-03	2.047E-03
rs3871466	6	31091662	G	0.430	2.033 [1.359-3.041]	0.232	0.129	4.079E-04	5.736E-04
rs2523898	6	31101512	A	0.417	0.542 [0.383-0.766]	0.331	0.478	3.322E-04	4.728E-04
rs3130955	6	31162490	A	0.211	1.541 [1.109-2.141]	0.470	0.365	7.960E-03	9.743E-03

					Psoriatic Arthritis				
RS #	CHR	MapInfo (bp)	Minor Allele	HW P (controls)	OR	MAF(cases)	MAF(controls)	Trend P	Trend P (adj.)
rs2233956	6	31189184	G	0.879	1.867 [1.289-2.704]	0.292	0.181	8.866E-04	1.204E-03
rs3094211	6	31194381	G	0.904	0.439 [0.266-0.725]	0.125	0.245	9.825E-04	1.328E-03
rs3094205	6	31199841	G	0.838	1.492 [1.069-2.084]	0.417	0.324	1.719E-02	2.030E-02
rs3131003	6	31201461	A	0.928	1.668 [1.200-2.319]	0.565	0.438	2.174E-03	2.827E-03
rs3815087	6	31201566	A	0.443	1.993 [1.396-2.844]	0.367	0.225	1.057E-04	1.592E-04
rs746647	6	31222161	G	0.413	1.758 [1.262-2.449]	0.452	0.320	6.302E-04	8.719E-04
rs1265112	6	31225998	G	0.413	1.758 [1.262-2.449]	0.452	0.320	6.302E-04	8.719E-04
rs130065	6	31230479	A	0.295	2.139 [1.497-3.056]	0.378	0.221	4.190E-05	6.606E-05
rs3130453	6	31232828	G	0.284	0.490 [0.348-0.690]	0.339	0.512	2.629E-05	4.231E-05
rs720465	6	31233756	A	0.396	2.158 [1.549-3.006]	0.482	0.301	3.307E-06	5.895E-06
rs1419881	6	31238572	A	0.786	1.648 [1.185-2.291]	0.565	0.441	3.022E-03	3.871E-03
rs1265159	6	31248026	A	0.614	2.332 [1.659-3.279]	0.411	0.230	6.546E-07	1.267E-06
rs3130473	6	31307187	A	0.573	2.308 [1.653-3.222]	0.464	0.273	1.162E-06	2.181E-06
rs3130685	6	31314185	A	0.786	1.563 [1.123-2.175]	0.554	0.443	8.230E-03	1.005E-02
rs2394963	6	31359441	A	0.144	0.364 [0.224-0.593]	0.120	0.273	4.883E-05	7.640E-05
rs3873379	6	31370148	G	0.177	0.379 [0.235-0.611]	0.125	0.274	6.987E-05	1.075E-04
rs2894207	6	31371730	G	0.895	1.903 [1.338-2.705]	0.345	0.217	2.819E-04	4.038E-04
rs9366778	6	31377152	A	0.136	0.427 [0.290-0.628]	0.220	0.398	2.049E-05	3.345E-05
rs10484554	6	31382534	A	0.234	2.526 [1.752-3.643]	0.321	0.158	5.794E-07	1.126E-06
rs2523619	6	31426123	G	0.809	1.633 [1.152-2.316]	0.345	0.244	5.514E-03	6.863E-03
rs2596503	6	31428789	A	1.000	0.408 [0.216-0.772]	0.073	0.162	4.771E-03	5.981E-03
rs2523608	6	31430538	G	0.565	1.505 [1.076-2.106]	0.469	0.369	1.479E-02	1.759E-02
rs7743761	6	31444079	A	0.718	1.703 [1.204-2.408]	0.357	0.246	2.980E-03	3.818E-03
rs2523535	6	31444229	G	0.781	0.691 [0.487-0.980]	0.319	0.404	3.611E-02	4.122E-02
rs2844529	6	31461572	A	0.433	1.564 [1.123-2.179]	0.458	0.351	9.243E-03	1.123E-02
rs2428486	6	31462083	G	0.433	1.605 [1.154-2.232]	0.464	0.351	5.634E-03	7.006E-03
rs13437082	6	31462539	A	0.642	2.057 [1.465-2.889]	0.416	0.257	3.146E-05	5.016E-05
rs4711269	6	31462798	A	0.642	2.116 [1.511-2.965]	0.423	0.257	1.345E-05	2.233E-05
rs2523467	6	31470909	A	0.327	1.523 [1.091-2.126]	0.451	0.351	1.527E-02	1.812E-02
rs1051794	6	31487088	A	0.408	1.608 [1.152-2.246]	0.423	0.313	5.668E-03	7.047E-03
rs1131896	6	31487094	A	0.103	0.363 [0.218-0.604]	0.112	0.259	1.210E-04	1.806E-04
rs7772549	6	31515622	G	0.264	1.576 [1.126-2.205]	0.433	0.326	9.089E-03	1.106E-02
rs9469003	6	31515807	G	0.281	1.882 [1.270-2.789]	0.244	0.146	1.839E-03	2.411E-03
rs2596480	6	31533964	A	0.606	0.287 [0.115-0.716]	0.030	0.097	3.851E-03	4.875E-03
rs2395029	6	31539759	C	1.000	2.813 [1.523-5.194]	0.095	0.036	4.311E-04	6.067E-04

					Psoriatic Arthritis				
RS #	CHR	MapInfo (bp)	Minor Allele	HW P (controls)	OR	MAF(cases)	MAF(controls)	Trend P	Trend P (adj.)
rs2248462	6	31554775	A	0.901	1.574 [1.102-2.247]	0.325	0.234	1.216E-02	1.459E-02
rs2516509	6	31557973	G	0.901	1.563 [1.092-2.237]	0.323	0.234	1.434E-02	1.707E-02
rs2844509	6	31618903	G	0.738	1.341 [0.947-1.899]	0.339	0.277	1.020E-01	1.111E-01
rs9368699	6	31910520	G	1.000	2.021 [1.057-3.865]	0.077	0.040	2.638E-02	3.054E-02
rs12198173	6	32134786	A	0.594	1.983 [1.253-3.137]	0.167	0.092	3.220E-03	4.113E-03
rs13199524	6	32174743	A	0.571	1.961 [1.222-3.146]	0.158	0.088	5.155E-03	6.439E-03
rs12153855	6	32182782	G	0.819	1.877 [1.211-2.909]	0.184	0.108	4.223E-03	5.325E-03
rs3096697	6	32242488	A	0.194	1.997 [1.407-2.834]	0.361	0.221	7.129E-05	1.091E-04
rs3134945	6	32254470	A	0.156	1.979 [1.394-2.808]	0.361	0.222	8.533E-05	1.295E-04
rs8192591	6	32293774	A	1.000	2.451 [1.292-4.651]	0.084	0.036	3.831E-03	4.852E-03
rs2395185	6	32541145	A	0.832	1.541 [1.098-2.163]	0.398	0.300	1.170E-02	1.406E-02
rs560788	6	52938820	A	0.523	1.710 [1.231-2.376]	0.554	0.420	1.321E-03	1.762E-03
rs10484710	6	56213713	A	0.110	N.D.	0.000	0.062	2.284E-03	2.964E-03
rs346298	6	80630001	A	0.075	2.127 [1.508-3.000]	0.387	0.229	3.226E-05	5.143E-05
rs7754913	6	132423548	C	0.689	2.209 [1.484-3.289]	0.244	0.127	8.445E-05	1.282E-04
rs12182899	6	148982366	G	0.904	0.840 [0.566-1.248]	0.214	0.245	3.818E-01	3.943E-01
rs6557200	6	150314225	G	0.108	1.959 [1.325-2.896]	0.253	0.147	3.647E-04	5.181E-04
rs4719601	7	2610133	A	0.541	0.402 [0.261-0.619]	0.161	0.323	2.282E-05	3.697E-05
rs10244600	7	16206679	C	0.085	1.284 [0.907-1.819]	0.354	0.299	1.798E-01	1.914E-01
rs1404398	7	16628533	A	0.292	2.323 [1.592-3.391]	0.289	0.149	1.715E-05	2.823E-05
rs156301	7	24190049	A	0.312	0.507 [0.330-0.779]	0.167	0.283	2.124E-03	2.766E-03
rs12535722	7	129259292	A	1.000	N.D.	0.000	0.084	3.278E-04	4.680E-04
rs1484646	8	10065806	A	0.202	0.605 [0.405-0.903]	0.207	0.302	1.041E-02	1.258E-02
rs4150895	8	86292487	A	0.619	N.D.	0.000	0.048	5.161E-03	6.446E-03
rs39519	8	90915209	A	1.000	1.673 [1.205-2.324]	0.494	0.368	2.020E-03	2.636E-03
rs1827656	8	122260569	A	1.000	2.791 [1.662-4.686]	0.137	0.054	5.020E-05	7.834E-05
rs1847547	8	122260683	A	1.000	2.940 [1.726-5.009]	0.132	0.049	3.422E-05	5.434E-05
rs4733741	8	130857730	A	0.701	0.525 [0.359-0.768]	0.232	0.365	8.315E-04	1.132E-03
rs7024449	9	691026	A	0.369	0.402 [0.262-0.615]	0.169	0.336	3.048E-05	4.867E-05
rs4740820	9	5600381	A	1.000	0.862 [0.588-1.263]	0.238	0.266	4.417E-01	4.537E-01
rs7042098	9	27815454	A	0.447	1.014 [0.708-1.453]	0.292	0.289	9.402E-01	9.418E-01
rs823918	9	103703117	C	0.069	1.596 [1.041-2.447]	0.190	0.128	3.851E-02	4.384E-02
rs10793992	9	133139159	A	0.194	0.683 [0.482-0.966]	0.321	0.410	2.616E-02	3.029E-02
rs10901365	9	133150374	A	0.263	0.627 [0.437-0.899]	0.296	0.402	8.694E-03	1.059E-02
rs1382580	10	23372821	G	0.358	0.223 [0.097-0.513]	0.036	0.142	1.013E-04	1.529E-04

					Psoriatic Arthritis				
RS #	CHR	MapInfo (bp)	Minor Allele	HW P (controls)	OR	MAF(cases)	MAF(controls)	Trend P	Trend P (adj.)
rs384037	11	2869129	G	0.083	0.474 [0.329-0.683]	0.262	0.428	3.053E-05	4.892E-05
rs1365057	11	35066251	A	0.793	0.892 [0.592-1.343]	0.196	0.215	5.804E-01	5.902E-01
rs11605924	11	45829667	A	0.204	0.564 [0.399-0.797]	0.362	0.502	1.504E-03	1.995E-03
rs1447170	11	57632797	G	0.279	2.258 [1.375-3.711]	0.143	0.069	1.191E-03	1.598E-03
rs10736719	11	61756322	G	0.410	1.328 [0.946-1.862]	0.381	0.317	9.727E-02	1.063E-01
rs3850942	11	63364623	G	0.106	3.085 [1.824-5.217]	0.137	0.049	1.410E-05	2.336E-05
rs495366	11	102200318	A	0.477	1.524 [1.074-2.162]	0.339	0.252	1.859E-02	2.187E-02
rs2661969	11	134223427	G	0.895	1.154 [0.784-1.697]	0.238	0.213	4.730E-01	4.846E-01
rs1040092	12	58052436	A	0.823	2.282 [1.513-3.441]	0.226	0.113	5.871E-05	9.108E-05
rs7957738	12	58057308	A	1.000	2.339 [1.562-3.501]	0.241	0.119	2.161E-05	3.517E-05
rs1607784	12	76246319	A	0.456	1.808 [1.147-2.851]	0.167	0.100	9.265E-03	1.126E-02
rs995030	12	87414802	A	0.337	1.876 [1.286-2.737]	0.274	0.167	1.111E-03	1.494E-03
rs1022034	12	87442874	A	0.337	1.933 [1.328-2.814]	0.280	0.167	5.805E-04	8.034E-04
rs3782181	12	87477692	C	0.444	2.037 [1.411-2.939]	0.304	0.176	1.451E-04	2.155E-04
rs3886972	12	92161554	A	0.245	1.512 [0.609-3.757]	0.036	0.024	3.795E-01	3.920E-01
rs10840624	12	120785682	G	0.703	1.792 [1.288-2.493]	0.518	0.375	4.773E-04	6.684E-04
rs2760908	13	26844485	G	0.897	2.015 [1.422-2.854]	0.363	0.221	6.412E-05	9.870E-05
rs12864419	13	28053904	C	0.717	0.673 [0.478-0.947]	0.345	0.439	2.437E-02	2.832E-02
rs1186468	13	38847162	A	0.545	0.638 [0.437-0.932]	0.238	0.329	1.871E-02	2.201E-02
rs4514547	13	39136899	G	0.923	0.631 [0.436-0.914]	0.259	0.357	1.465E-02	1.743E-02
rs4569133	13	39137044	A	0.923	0.621 [0.429-0.899]	0.256	0.357	1.138E-02	1.369E-02
rs7993214	13	39248912	A	0.629	0.627 [0.435-0.906]	0.262	0.361	1.381E-02	1.647E-02
rs9533755	13	43530210	G	0.725	1.701 [0.996-2.907]	0.113	0.070	5.122E-02	5.756E-02
rs288726	13	106292420	A	1.000	2.094 [1.462-2.998]	0.327	0.189	4.822E-05	7.526E-05
rs8018041	14	24520721	G	0.850	0.755 [0.448-1.272]	0.108	0.139	2.879E-01	3.007E-01
rs1958589	14	33914127	G	0.406	0.735 [0.418-1.291]	0.091	0.120	2.702E-01	2.828E-01
rs847506	14	35538760	A	1.000	1.865 [1.125-3.094]	0.132	0.076	1.214E-02	1.457E-02
rs7153045	14	55911652	G	0.097	1.784 [1.280-2.487]	0.494	0.354	3.582E-04	5.076E-04
rs1189046	14	55929403	A	0.464	0.484 [0.336-0.699]	0.262	0.423	7.182E-05	1.102E-04
rs2128673	14	56358349	A	0.271	1.780 [1.156-2.743]	0.190	0.117	8.574E-03	1.046E-02
rs431649	14	67647645	G	0.033	1.127 [0.745-1.704]	0.196	0.178	5.881E-01	5.978E-01
rs372143	14	67657091	G	0.033	1.127 [0.745-1.704]	0.196	0.178	5.881E-01	5.978E-01
rs2588833	14	67669245	A	0.065	1.134 [0.750-1.715]	0.196	0.177	5.655E-01	5.756E-01
rs2588832	14	67672520	C	0.076	1.207 [0.808-1.804]	0.214	0.184	3.756E-01	3.881E-01
rs1742501	14	68398902	G	0.735	3.242 [2.075-5.066]	0.202	0.073	1.275E-07	2.676E-07

					Psoriatic Arthritis				
RS #	CHR	MapInfo (bp)	Minor Allele	HW P (controls)	OR	MAF(cases)	MAF(controls)	Trend P	Trend P (adj.)
rs17126387	14	89371067	C	1.000	3.078 [1.657-5.720]	0.100	0.035	1.896E-04	2.775E-04
rs6575501	14	94710082	A	1.000	1.499 [0.780-2.882]	0.071	0.049	2.170E-01	2.292E-01
rs11637031	15	48576417	G	0.737	1.886 [1.285-2.767]	0.262	0.158	9.209E-04	1.248E-03
rs8037553	15	48577525	G	1.000	1.976 [1.345-2.905]	0.262	0.152	4.248E-04	5.975E-04
rs4775912	15	49068271	G	0.856	2.301 [1.574-3.365]	0.280	0.144	1.346E-05	2.244E-05
rs3803369	15	49163121	A	0.855	2.320 [1.586-3.393]	0.280	0.143	1.138E-05	1.914E-05
rs893576	15	49224953	G	1.000	2.327 [1.586-3.414]	0.274	0.139	9.291E-06	1.576E-05
rs4775919	15	49233201	G	1.000	2.397 [1.637-3.510]	0.280	0.139	3.764E-06	6.673E-06
rs8041933	15	49405038	A	0.880	2.059 [1.431-2.960]	0.315	0.183	8.755E-05	1.328E-04
rs288579	16	61135127	G	0.323	2.353 [1.538-3.600]	0.208	0.101	1.070E-04	1.608E-04
rs12447462	16	63038923	G	0.805	1.886 [1.202-2.959]	0.173	0.100	5.091E-03	6.361E-03
rs7193343	16	71586661	A	1.000	0.271 [0.136-0.542]	0.055	0.176	8.318E-05	1.269E-04
rs8059522	16	78014317	G	0.597	1.779 [1.198-2.641]	0.238	0.149	3.800E-03	4.814E-03
rs4782905	16	82813923	A	0.744	0.459 [0.182-1.155]	0.035	0.074	9.134E-02	1.000E-01
rs2240601	17	53106111	A	0.065	1.134 [0.750-1.715]	0.196	0.177	5.613E-01	5.715E-01
rs1941384	18	23988934	A	0.856	2.234 [1.524-3.273]	0.274	0.144	3.472E-05	5.516E-05
rs713042	19	1308082	A	0.228	1.761 [1.208-2.566]	0.280	0.181	2.477E-03	3.203E-03
rs3826942	19	1311575	G	0.532	1.755 [1.236-2.490]	0.345	0.231	1.367E-03	1.818E-03
rs8109578	19	10074154	A	1.000	0.108 [0.026-0.443]	0.013	0.108	1.989E-04	2.904E-04
rs1629174	19	44578058	A	1.000	1.529 [0.773-3.023]	0.065	0.044	2.168E-01	2.290E-01
rs2210455	20	9867383	A	1.000	2.091 [1.450-3.015]	0.309	0.176	7.201E-05	1.102E-04
rs6089151	20	30080496	A	0.711	N.D.	0.000	0.063	1.086E-03	1.464E-03
rs6057638	20	30802993	A	0.342	1.110 [0.727-1.695]	0.184	0.169	6.260E-01	6.349E-01
rs6141829	20	30902870	G	0.393	1.057 [0.677-1.651]	0.161	0.153	8.053E-01	8.103E-01
rs2000291	20	30923196	A	0.582	1.102 [0.700-1.736]	0.155	0.142	6.711E-01	6.792E-01
rs1936307	20	30993189	A	0.245	1.073 [0.741-1.553]	0.284	0.270	7.165E-01	7.235E-01
rs2827771	21	23231640	G	1.000	2.217 [1.481-3.318]	0.241	0.125	8.398E-05	1.276E-04
rs2829505	21	25307597	A	0.848	1.684 [1.213-2.339]	0.500	0.372	1.892E-03	2.477E-03
rs3746887	21	39954610	A	0.786	0.502 [0.304-0.828]	0.116	0.207	6.410E-03	7.925E-03
rs2041629	22	15937144	A	0.370	2.054 [1.429-2.951]	0.315	0.183	9.347E-05	1.418E-04
rs433576	22	16828960	A	0.114	0.899 [0.595-1.358]	0.223	0.242	6.279E-01	6.369E-01
rs390407	22	21588231	G	0.340	0.925 [0.631-1.357]	0.238	0.252	6.838E-01	6.916E-01
rs740234	22	29338745	G	0.869	1.261 [0.832-1.912]	0.196	0.162	2.662E-01	2.789E-01
rs5916793	X	107034204	C	1.000	3.695 [1.293-10.550]	0.044	0.012	2.946E-05	4.723E-05
rs4933045	XY	214201	A	0.392	1.182 [0.829-1.685]	0.337	0.301	3.601E-01	3.727E-01
