# Supplementary figures and images for: A Genome-Wide Association Study of Psoriasis and Psoriatic Arthritis Identifies New Disease Loci
Source: PLoS Genet. 2008 Apr 4;4(4):e1000041. doi: 10.1371/journal.pgen.1000041 (PMC2274885; doi:10.1371/journal.pgen.1000041)

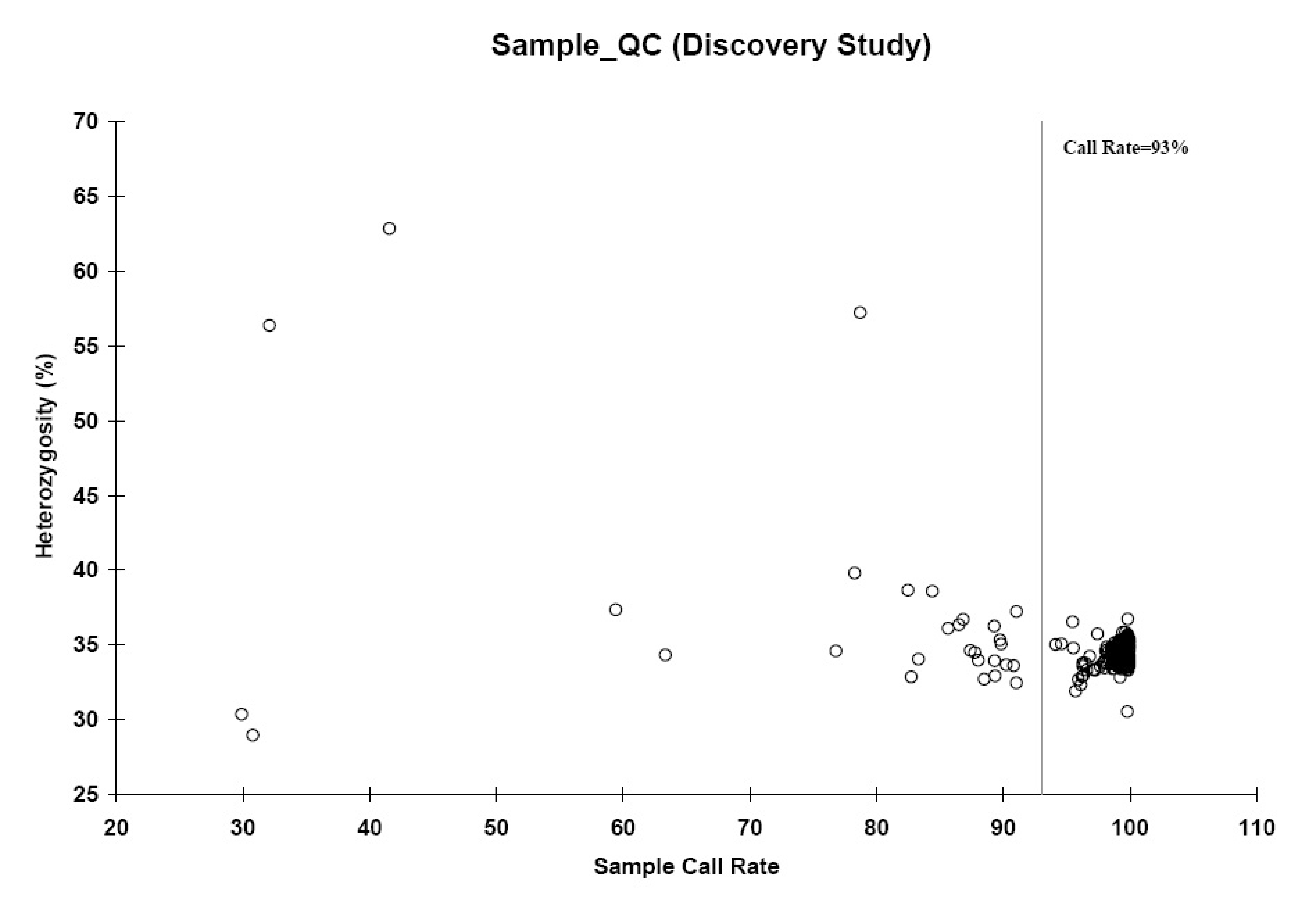

Supplement: Figure S1 — Heterozygosity of sample versus genotyping call rate. (0.95 MB TIF) [file pgen.1000041.s003.tif]

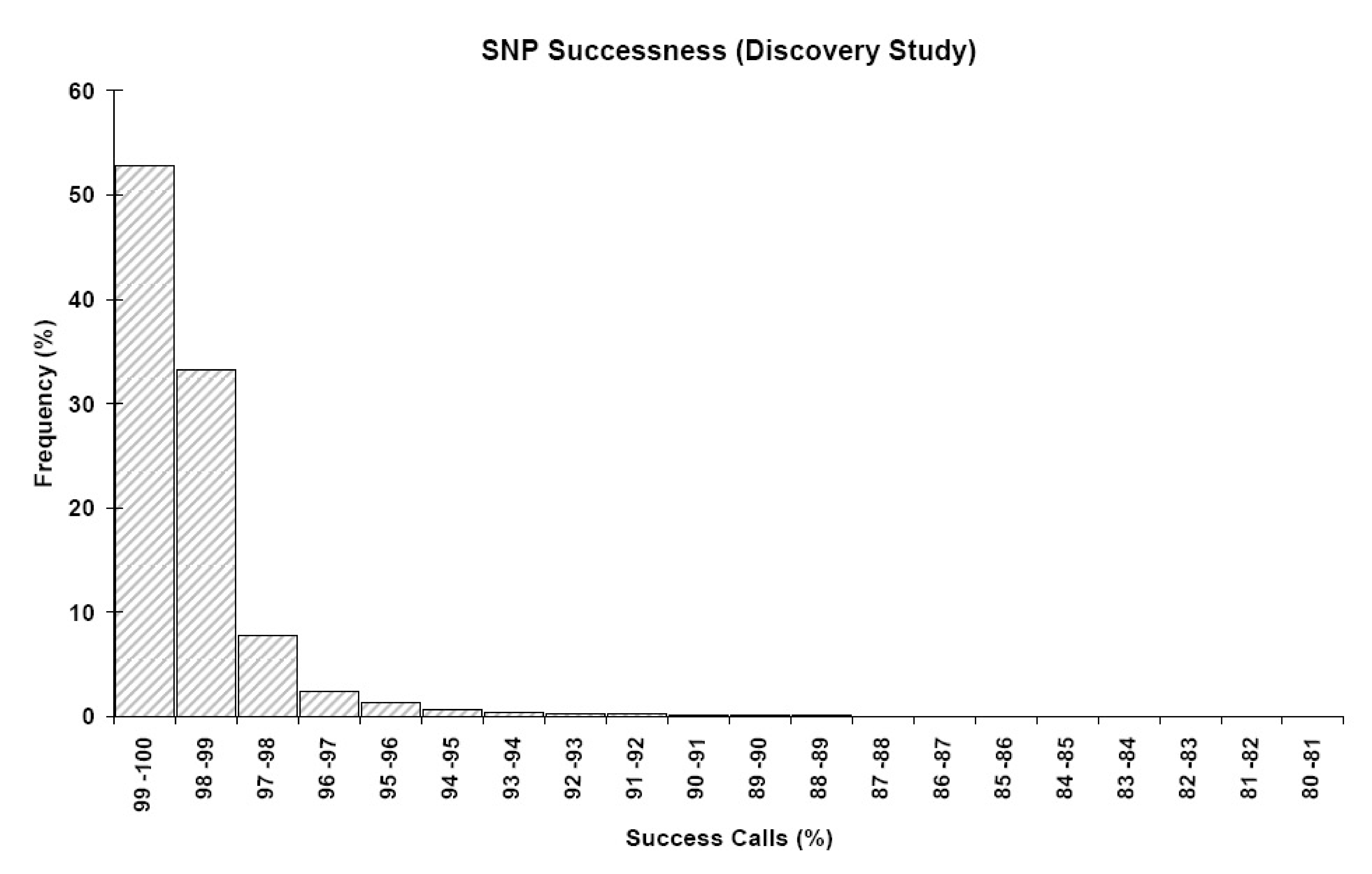

Supplement: Figure S2 — Distribution of SNP success rate in the discovery study. (1.36 MB TIF) [file pgen.1000041.s004.tif]
